# Identification of microRNAs regulated by tobacco curly shoot virus co-infection with its betasatellite in *Nicotiana benthamiana*

**DOI:** 10.1186/s12985-019-1234-5

**Published:** 2019-11-07

**Authors:** Jiang Du, Gentu Wu, Zhongpiao Zhou, Jiayuan Zhang, Mingjun Li, Miao Sun, Kairong Jiang, Ling Qing

**Affiliations:** Chongqing Key Laboratory of Plant Disease Biology, College of Plant Protection, Southwest University, Chongqing, 400716 the, People’s Republic of China

**Keywords:** MicroRNA, Tobacco curly shoot virus, Betasatellite, *Nicotiana benthamiana*, Small RNA-sequencing

## Abstract

**Background:**

MicroRNAs (miRNAs) are a class of 21–24 nucleotide endogenous non-coding small RNAs that play important roles in plant development and defense responses to biotic and abiotic stresses. Tobacco curly shoot virus (TbCSV) is a monopartite *begomovirus*, cause leaf curling and plant stunting symptoms in many Solanaceae plants. The betasatellite of TbCSV (TbCSB) induces more severe symptoms and enhances virus accumulation when co-infect the plants with TbCSV.

**Methods:**

In this study, miRNAs regulated by TbCSV and TbCSB co-infection in *Nicotiana benthamiana* were characterized using high-throughput sequencing technology.

**Results:**

Small RNA sequencing analysis revealed that a total of 13 known miRNAs and 42 novel miRNAs were differentially expressed in TbCSV and TbCSB co-infected *N. benthamiana* plants. Several potential miRNA-targeted genes were identified through data mining and were involved in both catalytic and metabolic processes, in addition to plant defense mechanisms against virus infections according to Gene Ontology (GO) analyses. In addition, the expressions of several differentially expressed miRNAs and their miRNA-targeted gene were validated through quantitative real time polymerase chain reaction (qRT-PCR) approach.

**Conclusions:**

A large number of miRNAs are identified, and their target genes, functional annotations also have been explored. Our results provide the information on *N. benthamiana* miRNAs and would be useful to further understand miRNA regulatory mechanisms after TbCSV and TbCSB co-infection.

## Background

MicroRNAs (miRNAs) are a large group of small, non-coding RNAs that are 21–24 nucleotides (nt) long and regulate gene expression at the post-transcriptional level [1, 2]. In plants, miRNA is initially produced by RNA polymerase II (Pol II) or III (Pol III) and is known as the primary miRNA (pri-miRNA). Then the pri-miRNA is processed by a Dicer-like (DCL) enzyme, which produces an miRNA precursor (pre-miRNA), which contains both a miRNA:miRNA* intermediate duplex and a stem-loop-like structure [3]. After excision, the mature miRNA is bound to an argonaute (AGO) protein to form a RNA-induced silencing complex (RISC) [4]. Finally, the RISC complex recognizes and binds with the target RNA transcript, in a sequence-specific manner, and then cleaves the transcript or represses the translation process [2].

Since the first report of plant miRNA in *Arabidopsis thaliana* [5, 6], numerous studies have shown that plant miRNAs play critical roles in regulating plant development [7, 8], gene translational repression [9], flowering and sex determination [10], phytohormone signaling [11], and plant responses to abiotic and/or biotic stresses [12] such as drought stress [13] and infections with viruses or other pathogens [14–16].

In addition, many studies have indicated that miRNAs are highly conserved in eukaryotes, and virus infections in plants often alter miRNA expressions. For example, tobacco mosaic virus (TMV) infection in *Nicotiana tabacum* changes the expressions of several specific miRNAs [17]. Infection of cotton plants with cotton leaf roll dwarf virus (CLRDV) affects miRNA expression, downregulating the expression of specific miRNAs and causing disease symptoms in cotton leaves [18]. In *A. thaliana* and *N. benthamiana* plants, the expressions of miR168 and the *Argonaute 1* gene are upregulated after infection with several plant viruses [19]. The expression of miR159 is upregulated by cucumber green mottle mosaic virus (CGMMV) infection cucumber at 10 post-inoculation [20]. In rice, downregulation of osa-miR171b expression in plants infected with rice stripe virus (RSV) enhances RSV disease symptoms [21]. The expression of miR319 in rice is increased after infection with rice ragged stunt virus (RRSV), and upregulation of miR319 promotes RRSV infection and disease symptoms by inhibiting JA-mediated host defense [22]. Amin et al. (2011) reported that begomovirus infection upregulates the accumulation of miRNAs controlling plant development [23].

*Geminiviridae* has nine genera: *Mastrevirus*, *Curtovirus*, *Begomovirus*, *Topocuvirus*, *Becurtovirus*, *Turncurtovirus*, *Eragrovirus*, *Capulavir*, and *Euphorbia* [24]. The family *Geminiviridae* currently consists of 468 species, in which *Begomovirus* is the largest genus (https://talk.ictvonline.org/taxonomy/). *Begomovirus* has about 409 species or members that cause severe damage to economically important food crops [25–27]. TbCSV was initially identified in tobacco plants and is a monopartite begomovirus [28]. Monopartite begomoviruses often co-infect their host plants with specific betasatellite DNA [29]. Betasatellite DNA genomes are typically about half the size of begomovirus DNA genomes, and play essential roles in the symptoms induction by monopartite begomoviruses [30, 31]. Host plants infected with TbCSV often display clear disease symptoms [32, 33]. When host plants are co-infected with TbCSV and TbCSB (TbCSV/TbCSB), they often show enhanced disease symptoms [33, 34].

In this study, a miRNA-sequencing approach was used to identify *N. benthamiana* miRNAs regulated by TbCSV/TbCSB co-infection. Then we predicted the target genes of the differentially expressed miRNAs. To investigate the interactions between the differentially expressed miRNAs and their target genes during TbCSV/TbCSB co-infection in *N. benthamiana*, the expression levels of several identified miRNAs together with their target genes were analyzed using qRT-PCR. The results not only shed light on the possible roles of miRNAs in *N. benthamiana* development and physiology, but also their possible roles in *N. benthamiana* resistance to TbCSV/TbCSB co-infection.

## Methods

### Plant growth and virus inoculation

*N. benthamiana* plants were grown inside a greenhouse at 24 °C and a 16/8 h (light/dark) photoperiod. Infectious clones of TbCSV isolate Y35 and its betasatellite (TbCSB) were individually introduced to *Agrobacterium tumefaciens* strain EHA105 [33]. After overnight culturing and centrifugation, agrobacterium pellets were resuspended in infiltration buffer (10 mM MES, 10 mM MgCl_2_ and 200 μM acetosyringone in sterile water) until they reached an OD_600_ value of 2, followed by 3 h incubation at room temperature. The two agrobacterium cultures were mixed 1:1 (v/v) and then co-infiltrated into the leaves of *N. benthamiana* plants at the six to eight leaf stage as described previously [35]. Plants infiltrated with the infiltration buffer only were used as non-infected controls.

### Sample preparation and total RNA isolation

At 20 days-post co-infiltration (dpi), systemic leaves of three TbCSV/TbCSB-infected and three non-infected control plants were harvested. Total RNA was isolated from leaf samples using the TRIzol reagent, following the manufacturer’s instructions (Invitrogen, Waltham, MA, USA).

### Small RNA library construction and Illumina sequencing

The integrity and concentration of RNA samples were checked using a NanoDrop spectrophotometer and an Agilent 2100 Bioanalyzer, following the manufacturer’s instructions (Agilent, Santa Clara, CA, USA). High-quality samples were identified and used to construct small RNA libraries followed by Illumina sequencing using an IlluminaHiseq™2500 instrument. Small RNA libraries and Illumina sequencing were performed by the Novogene Bioinformatics Technology Company, Beijing, China.

### Analyses of Illumina sequencing date

Raw reads were processed to remove both adaptors and low-quality reads. Both the commercial GeneChip® Tomato Genome Array and the miRBase (http://www.mirbase.org/) were searched to identify known miRNAs in the two small RNA libraries (i.e., TbCSV/TbCSB and mock). The reads matched to the *N. benthamiana* genome shotgun-sequence assemblies were kept for further identifications.

### Prediction of miRNA-targeted genes and gene function analyses

Potential miRNA-targeted genes were predicted using the psRNATarget program (http://plantgrn.noble.org/psRNATarget). The rules used for the predictions were as described by Schwab et al. [36]. To explore the possible functions of the predicted target genes, Gene Ontology (GO) analyses were performed as described previously [37]. The results were split into three different categories: Biological process, Molecular functions and Cellular components. Through this process, all potential target genes were mapped to GO terms described in the database (http://www.geneontology.org).

### Validations of miRNA and target gene expression

qRT-PCR was performed to validate the expressions of miRNAs together with their target genes. Total RNA was isolated at 20 dpi from *N. benthamiana* leaves either infected with TbCSV/TbCSB or not, using TRIzol reagent. For miRNAs, the cDNA was synthesized by RT using the PrimeScript RT reagent Kit with gDNA Eraser with a special stem-loop RT primer according to the manufacturer’s instructions (TaKaRa, Dalian, China). The cDNA products were used as templates for real-time PCR analyses. The primers used for qRT-PCR assays are listed in (Additional file 1 Table S1). In addition, we also used qRT-PCR to analyze the expression of target genes, and the primers are also listed in Table S1. Purified total RNA was used as a template and reverse-transcribed using the PrimeScript RT reagent Kit (TaKaRa, Dalian, China) to obtain cDNA. The reactions consisted of incubation in 96-well plates at 98 °C for 2 min, followed by 40 cycles of 98 °C for 10 s, 60 °C for 10 s, and 68 °C for 30 s. The expression level of the *N. benthamiana Ubiquitin C* gene (UBC) was used as the reference [38, 39]. Three biological samples were used for each treatment and each biological sample had a further three technical replicates during qPCR. The qPCR results were calculated using the 2^-ΔΔCT^ method [38].

## Results

### Small RNA library construction

**Fig. 1:**
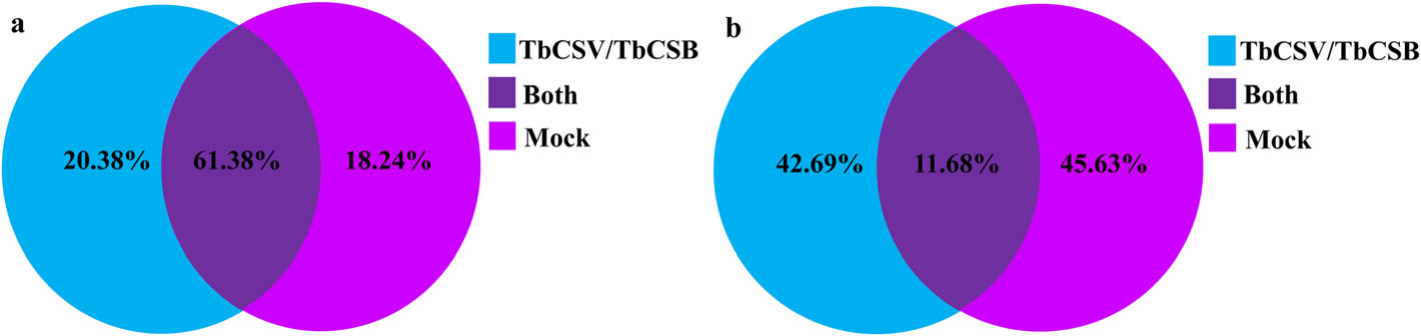
Percentages of the common and specific sequences of the clean reads (**a**) and unique sRNAs (**b**) from the TbCSV/TbCSB-infected and non-infected (Mock) control libraries

Small RNA libraries were constructed using total RNA from *N. benthamiana* leaves either infected with TbCSV/TbCSB or not. A total of 16,753,586 and 17,822,708 raw reads were generated via Illumina sequencing to represent these samples. After removing raw reads without 3′ adapters, with 5′ added adapters, with more than 10% unidentified nucleotides (nt), with poly (A/T/G/C) stretches, and that were less than 18 nt or more than 30 nt, a total of 13,896,237 and 15,227,477 clean reads were obtained from infected and non-infected libraries, respectively (Table 1). Of these, 5,936,641 (20.38%) were only in the infected library and 5,311,979 (18.24%) were only in the non-infected library (Fig. 1a). As shown in Fig. 1b, 10,438,652 unique reads were revealed, including 1,219,644 common reads and 44,562,19 and 47,627,89 specific reads for TbCSV/TbCSB and mock, respectively.

**Table 1:** Summary of read data produced by small RNA sequencing

sRNA	TbCSV/TbCSB	Mock
Total reads	16,753,586	17,822,708
Low quality reads	14,843	16,495
3′ adapter null or insert null reads	252,259	312,162
5′ adapter contaminants reads	3772	9622
N% > 10%	1274	1347
With ployA/T/G/C reads	58,177	63,927
< 18 nt, > 30 nt	2,527,024	2,191,678
Clean reads	13,896,237	15,227,477
sRNAs mapping to genome	10,080,639	12,671,385
Unique reads mapping to genome	3,745,058	4,241,949

The resulting clean small RNA reads belonged to different categories, including exon sense and antisense, intron sense and antisense, rRNA, tRNA, snRNA, snoRNA, RNA repeats, natural antisense transcripts (NAT), trans-acting siRNA (TAS), and other unannotated reads. Among these, there were 404,032 (4.01%) miRNA tags from the infected library and 408,746 (3.22%) from the non-infected library (Table 2).

**Table 2:** Distribution of small RNA sequences among the two constructed libraries

	Mock		TbCSV/TbCSB	
	Total sRNA reads (percent %)	Unique sRNA reads (percent %)	Total sRNA reads (percent %)	Unique sRNA reads (percent %)
Total	12,671,385 (100%)	4,241,949 (100%)	10,080,639 (100%)	3,745,058 (100%)
Exon sense	470,589 (3.71%)	170,880 (4.03%)	369,364 (3.66%)	119,349 (3.19%)
Exon antisense	567,077 (4.48%)	44,166 (1.04%)	387,710 (3.85%)	44,418 (1.19%)
Intron sense	1,057,103 (8.34%)	363,908 (8.58%)	975,273 (9.67%)	349,541 (9.33%)
Intron antisense	675,481 (5.33%)	198,623 (4.68%)	505,824 (5.02%)	179,369 (4.79%)
miRNA	408,746 (3.22%)	1501 (0.04%)	404,032 (4.01%)	1488 (0.04%)
rRNA	638,241 (5.04%)	31,900 (0.75%)	443,250 (4.4%)	27,223 (0.73%)
tRNA	1 (0%)	1 (0%)	1 (0%)	1 (0%)
snRNA	3576 (0.03%)	841 (0.02%)	3584 (0.04%)	1054 (0.03%)
snoRNA	26,219 (0.21%)	1688 (0.04%)	17,338 (0.17%)	1678 (0.04%)
Repeat	3,991,675 (31.5%)	801,555 (18.9%)	2,985,768 (29.62%)	741,474 (19.8%)
NAT	297,399 (2.35%)	9280 (0.22%)	186,878 (1.85%)	7343 (0.2%)
TAS	15,340 (0.12%)	688 (0.02%)	15,305 (0.15%)	704 (0.02%)
Unannotated	4,519,938 (35.67%)	2,616,918 (61.69%)	3,786,312 (37.56%)	2,271,416 (60.65%)

**Fig. 2:**
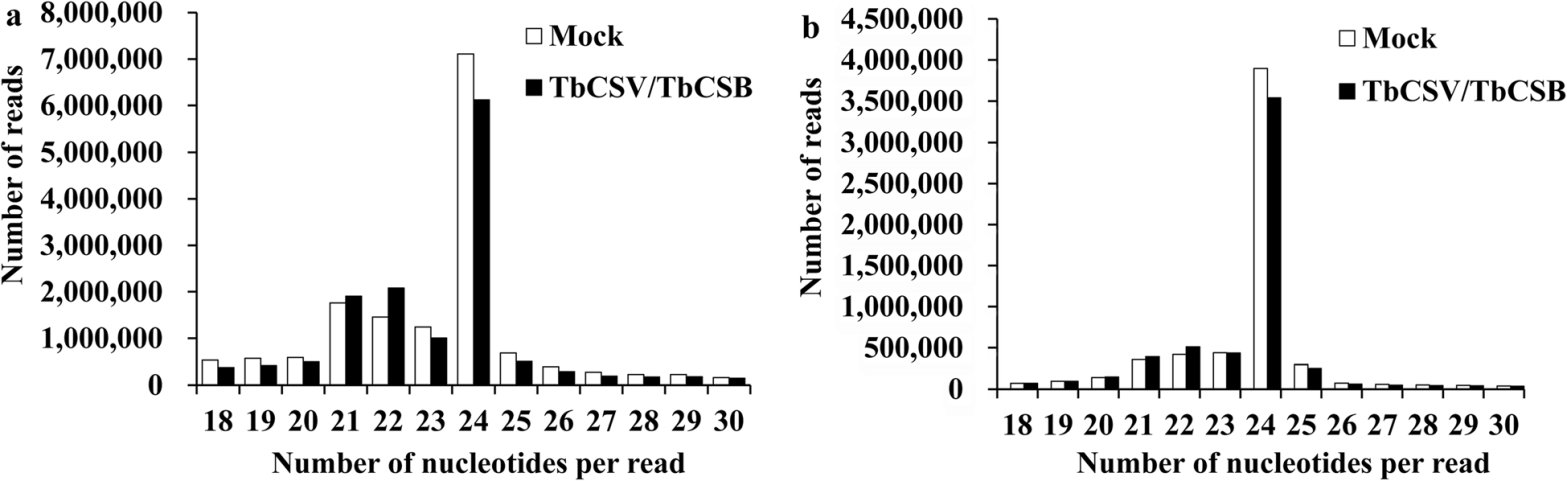
Numbers of total miRNA reads (**a**) and unique reads (**b**) with specific numbers of nucleotides from the TbCSV/TbCSB-infected and non-infected (Mock) *N. benthamiana* libraries

When all of the reads (18 to 30 nt) were analyzed, those with 24 nt were the most abundant. Of these, 6,119,284 (44.04%) were found in the infected library and 7,109,221 (46.69%) were found in the non-infected library. The second most abundant reads (1,760,916 reads or 11.56%) in the non-infected library had 21 nt, and that (2,079,345 reads or 14.96%) in the infected library had 22 nt (Fig. 2 a). When only unique reads were considered, 24 nt reads were the most abundant class with a total of 3,538,301 reads (62.34%) in the infected library and 3,896,777 reads (65.14%) in the non-infected library. The second most abundant unique read (510,867 reads or 9.0%) in the infected library had 22 nt while that (438,923 reads or 7.34%) in the non-infected library had 23 nt (Fig. 2b).

### Identification of known miRNAs

**Fig. 3:**
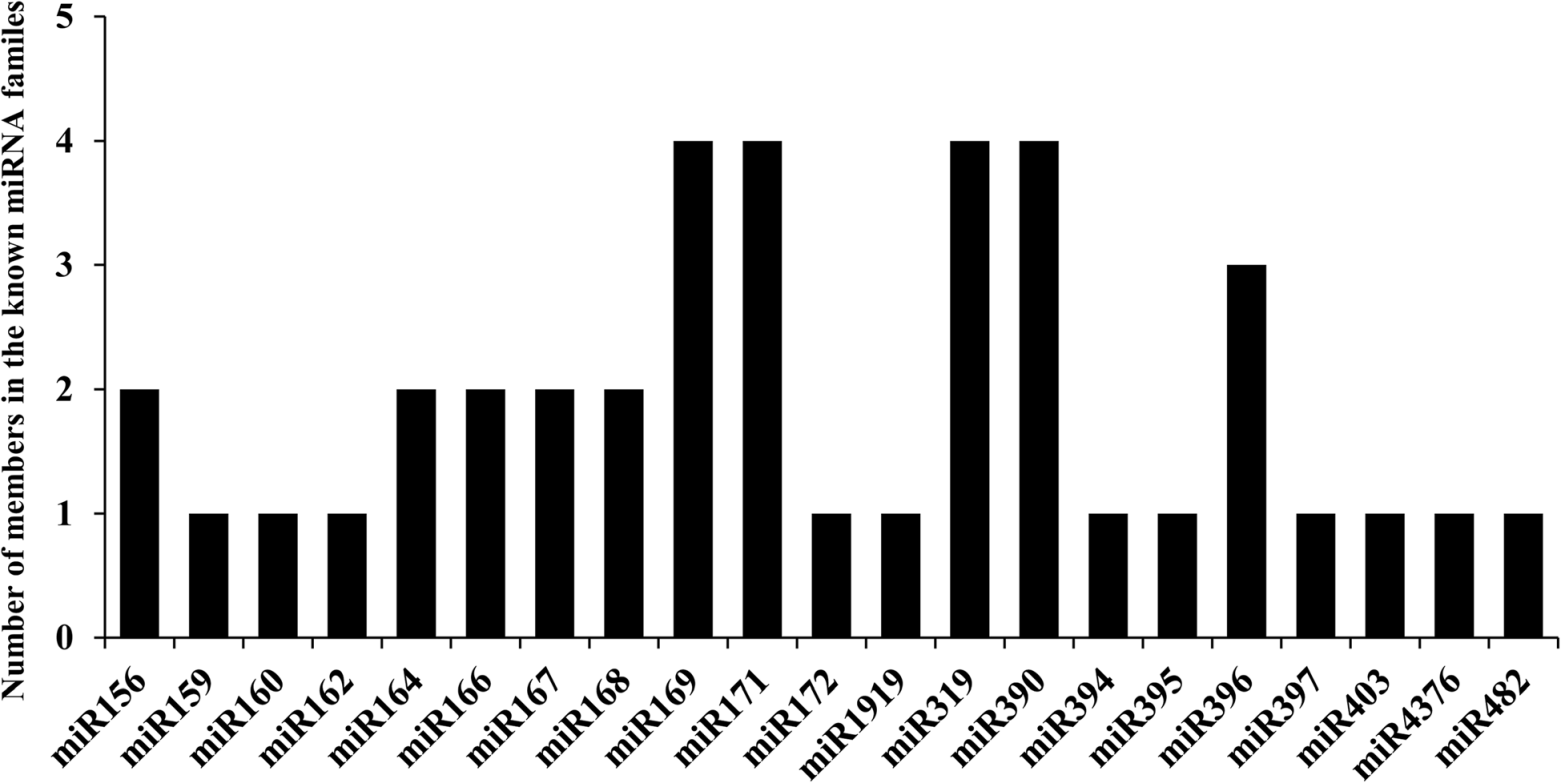
Numbers of miRNA members identified in each conserved miRNA family in the small RNA libraries

**Fig. 4:**
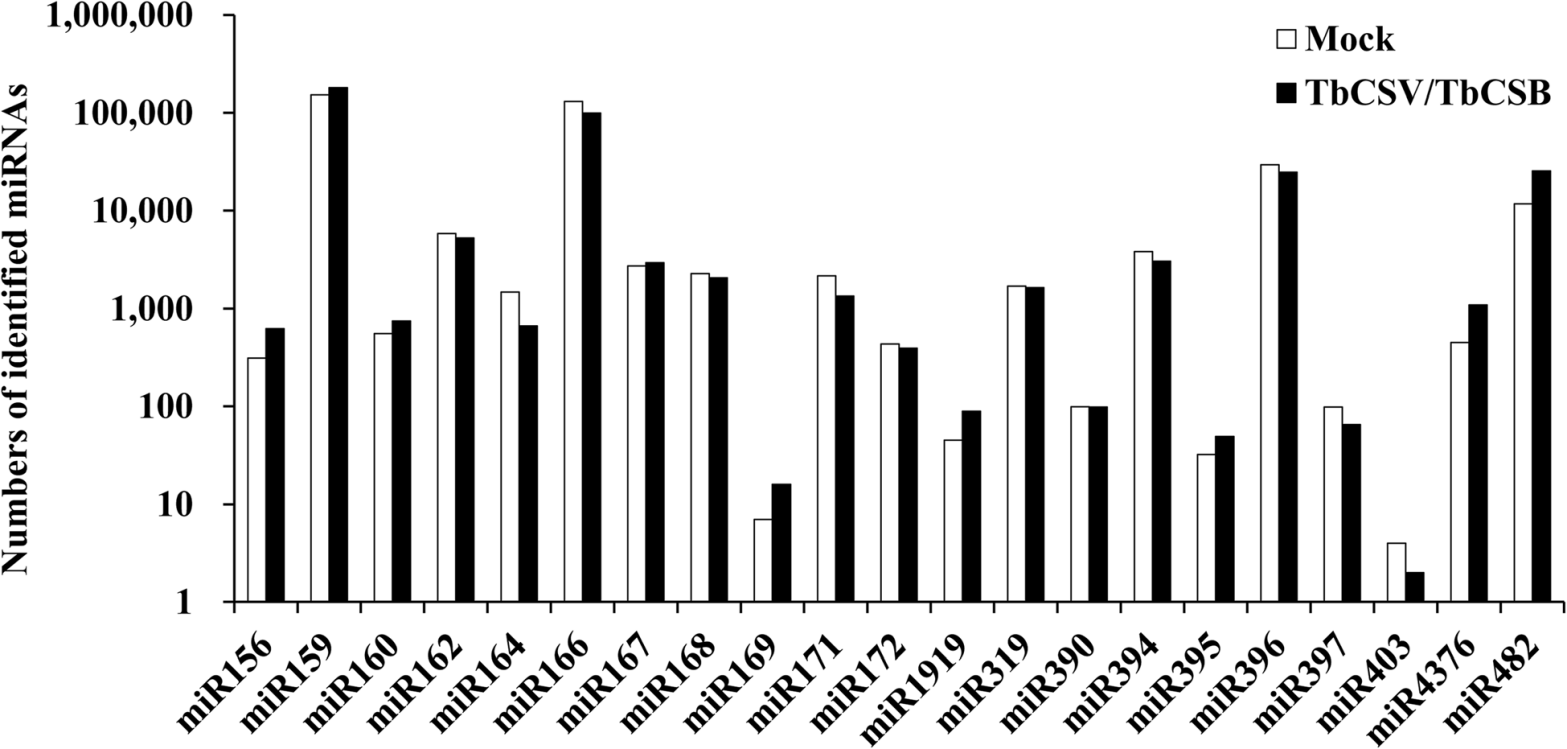
Numbers of identified miRNAs in each conserved miRNA family from the TbCSV/TbCSB-infected and non-infected (Mock) libraries

To identify known miRNAs in the two libraries, all small RNA reads were used to blast search the miRBase site with known mature plant miRNAs and then the *N. benthamiana* genome database. Through this approach, a total of 349,937 small RNA reads from the infected library and 346,265 reads from the non-infected library were mapped to the *Solanum lycopersicum* genome. Excluding miRNAs expressed at extremely low levels, 40 miRNAs in 21 known miRNA families were identified. The number of small RNA reads matched to known miRNA families are summarized in Figs. 3 and 4. The expression levels of miRNAs changed slightly after infection, as shown in Table 3. There were 13 differentially expressed miRNAs within the two libraries, with 2 downregulated miRNAs and 11 upregulated miRNAs after TbCSV/TbCSB co-infection.

**Table 3:** miRNAs expressed differentially between the TbCSV/TbCSB and Mock

miRNA	TbCSV/TbCSB			Mock	Fold-change	*P*-value	Mode	Sig-lable
name	Counts	Normalized	Counts	Normalized	log_2_(TbCSV/TbCSB/ Mock)			
novel-10	887	2212.16	633	1561.89	0.61	2.29E-26	up	**
novel-103	75	187.05	55	135.71	0.57	0.0042	up	**
novel-104	27	67.34	44	108.57	−0.59	0.0018	down	**
novel-105	17	42.40	64	157.92	−1.79	3.96E-17	down	**
novel-112	2161	5389.50	1656	4086.07	0.50	3.20E-41	up	**
novel-113	9	22.45	24	59.22	−1.30	3.42E-05	down	**
novel-115	51	127.19	118	291.16	−1.09	4.40E-16	down	**
novel-121	56	139.66	21	51.82	1.53	9.87E-11	up	**
novel-127	118	294.29	249	614.39	−0.96	6.72E-27	down	**
novel-128	8	19.95	15	37.01	− 0.79	0.0227	down	*
novel-129	214	533.71	124	305.96	0.91	2.51E-15	up	**
novel-131	32	79.81	0	0.00	7.37	1.64E-19	up	**
novel-133	43	107.24	90	222.07	−0.95	1.59E-10	down	**
novel-137	23	57.36	46	113.50	−0.88	1.45E-05	down	**
novel-138	39	97.27	27	66.62	0.65	0.0163	up	*
novel-140	8	19.95	2	4.93	2.12	0.0019	up	**
novel-142	4	9.98	9	22.21	−1.05	0.0290	down	*
novel-15	723	1803.15	553	1364.49	0.51	5.49E-15	up	**
novel-16	348	867.91	745	1838.24	−0.98	1.73E-79	down	**
novel-18	320	798.07	218	537.90	0.67	8.97E-13	up	**
novel-21	365	910.30	265	653.87	0.58	7.87E-11	up	**
novel-31	244	608.53	176	434.27	0.59	6.23E-08	up	**
novel-32	113	281.82	239	589.72	−0.96	5.22E-26	down	**
novel-42	135	336.69	103	254.15	0.51	0.00067	up	**
novel-5	3355	8367.31	6145	15,162.39	−0.75	0	down	**
novel-50	104	259.37	67	165.32	0.75	4.54E-06	up	**
novel-52	64	159.61	115	283.76	−0.73	2.89E-09	down	**
novel-6	3704	9237.71	1040	2566.13	1.95	0	up	**
novel-60	19	47.39	34	83.89	−0.72	0.0013	down	**
novel-61	56	139.66	40	98.70	0.60	0.0078	up	**
novel-66	55	137.17	87	214.67	−0.54	3.32E-05	down	**
novel-69	6	14.96	39	96.23	−2.58	5.08E-16	down	**
novel-70	11	27.43	45	111.03	−1.91	2.03E-13	down	**
novel-71	45	112.23	16	39.48	1.61	1.64E-09	up	**
novel-75	21	52.37	38	93.76	−0.74	0.0006	down	**
novel-78	11	27.43	23	56.75	−0.95	0.0012	down	**
novel-82	14	34.92	8	19.74	0.93	0.0387	up	*
novel-9	687	1713.37	1732	4273.60	−1.22	1.30E-248	down	**
novel-91	29	72.33	14	34.54	1.17	0.0002	up	**
novel-92	45	112.23	82	202.33	−0.75	3.09E-07	down	**
novel-94	7	17.46	29	71.56	−1.93	2.99E-09	down	**
novel-96	1	2.49	4	9.87	−1.88	0.0302	down	*
miR156a	224	558.65	101	249.21	1.27	2.70E-28	up	**
miR156d-5p	401	1000.09	210	518.16	1.05	9.17E-36	up	**
miR160a	747	1863.01	554	1366.96	0.55	2.04E-18	up	**
miR164a-5p	655	1633.56	1469	3624.66	−1.05	1.32E-170	down	**
miR168b-3p	670	1670.97	477	1176.97	0.61	1.55E-20	up	**
miR169a	2	4.99	0	0.00	3.37	0.0423	up	*
miR169c	13	32.42	6	14.80	1.23	0.0094	up	**
miR171b	443	1104.83	1070	2640.16	−1.15	2.75E-143	down	**
miR1919c-5p	89	221.96	45	111.03	1.10	8.28E-10	up	**
miR390a-3p	2	4.99	0	0.00	3.37	0.0423	up	*
miR395a	49	122.21	32	78.96	0.73	0.0022	up	**
miR4376	1091	2720.94	451	1112.81	1.39	1.24E-153	up	**
miR482a	25,539	63,693.84	11,753	28,999.78	1.24	0	up	**

* represnt Fold-change (log_2_ TbCSV/TbCSB/Mock) > 1.0 or Fold-change (log_2_ TbCSV/TbCSB/Mock) < −1.0, and 0.01 < =P -values < 0.05; ** represnt Fold-change (log_2_ TbCSV/TbCSB/Mock) > 1.0 or Fold-change (log_2_ TbCSV/TbCSB/Mock) < − 1.0, and P -values < 0.01

### Identification of novel candidate miRNAs

To predict hairpin-like structures in the identified miRNA precursors and identify the corresponding miRNAs that could be used to further identify novel miRNAs, we utilized the miREvo and miRDeep2 software as described [40, 41]. We also used the mfold web server (http://unafold.rna.albany.edu) to predict the secondary structures and the minimum free energy of the annotated small RNA tags that were mapped to the *N. benthamiana* genome, as described previously [42]. After removing miRNAs with extremely low expressions, a total of 42 miRNAs were found to show differential expression between the two libraries. The novel-6 miRNA showed the highest abundance and had 9238 transcripts per million (TPM) followed by the novel-5 miRNA (8367 TPM) in the infected library. In the non-infected control library, the novel-5 miRNA showed the highest abundance (15,162 TPM) followed by the novel-9 miRNA (4273 TPM) (Table 3, Additional file 2 Figure S1). Among the predicted novel miRNA candidates, the base bias in the first position showed that the majority of these novel miRNA candidates started with a 5′ uridine (U) as shown in Fig. S2a and Fig. S2b. Furthermore, novel miRNA candidate nucleotide bias at each position were also analyzed (Additional file 3 Figure S2c and Figure S2d). In addition, we also found among the 42 differentially-expressed novel miRNAs, twenty-five had complementary miRNA*s, with precursor lengths ranging from 43 to 295 nt and predicted minimal folding energy (MFE) ranging from − 14.2 to − 130.7 kcal/mol. Also, 17 differentially-expressed novel miRNAs without miRNA*s detected were identified as candidate miRNAs (Additional file 4 Table S2). Fifteen out of 42 new miRNAs were 21 nt in length, while three, one, ten and thirteen miRNA had lengths of 19, 20, 22, and 24 nt, respectively (Additional file 4 Table S2). All of the novel miRNAs’ loci, pre-miRNA sequences and structures, and reads from deep sequencing were shown in Additional file 4. This is in agreement with published criteria for novel miRNA [43, 44], and suggests that these candidate miRNAs are most likely to be new miRNA family members in *N. benthamiana.*

### Prediction of potential miRNA-targeted genes

**Fig. 5:**
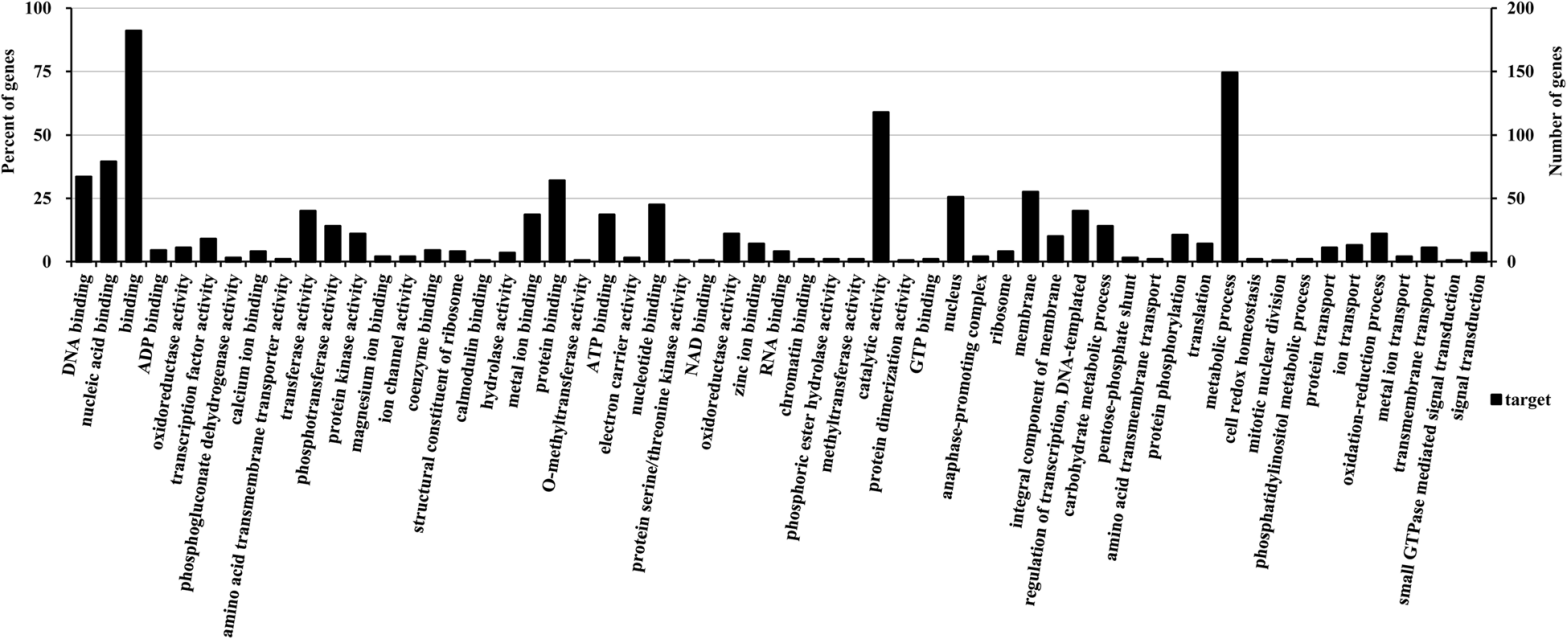
Gene ontology (GO) analysis using the predicted target genes regulated by the differentially expressed miRNAs

Numerous genes are responsive to virus infections and to differentially expressed miRNAs. Our results indicate that many potential miRNA-targeted genes encode transcription or non-transcription factor proteins, which are important for physiological processes. To explore the regulatory functions of the identified miRNAs in the infected library, potential target genes of nine conserved and four novel miRNAs were predicted by GO analyses. The GO annotated terms Biological process, Cellular components, and Molecular function were further analyzed to determine genes that could potentially be targeted by the identified miRNAs (Fig. 5). For the Biological process category, genes involved in metabolic processes (GO: 0008152) were the most represented GO terms. Membrane (GO: 0016020) and nucleus (GO: 0005634) were the major GO terms within the Cellular component category. For the Molecular function category, the major GO terms were binding (GO: 0005488) and catalytic activity (GO: 0003824). Many of the identified miRNA-targeted genes have previously been reported to play a role in defense against pathogens.

### Validations of miRNA and target gene expressions by qRT-PCR

**Fig. 6:**
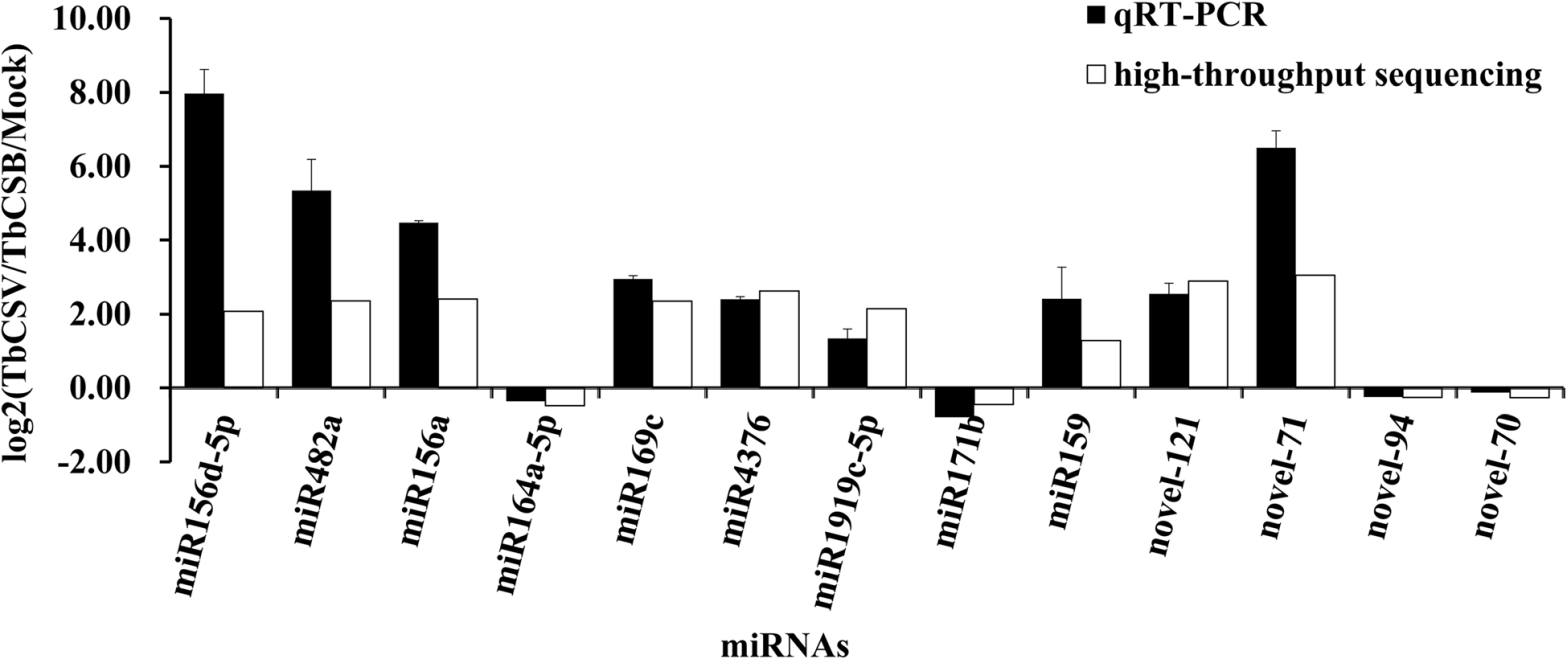
qRT-PCR and high-throughput sequencing analyses of the relative expressions of nine known and four new miRNAs. Total RNA was isolated from the TbCSV/TbCSB-infected or non-infected (Mock) *N. benthamiana* leaves. The x-axis shows the names of the miRNAs analyzed in this study. The y-axis shows the Log_2_ratio between the expression values from a TbCSV/TbCSB-infected sample versus its Mock sample. Three biological replicates were analyzed for each miRNA through qRT-PCR. Expression level of *N. benthamiana UBC* gene was used as the reference gene during qRT-PCR assays

**Fig. 7:**
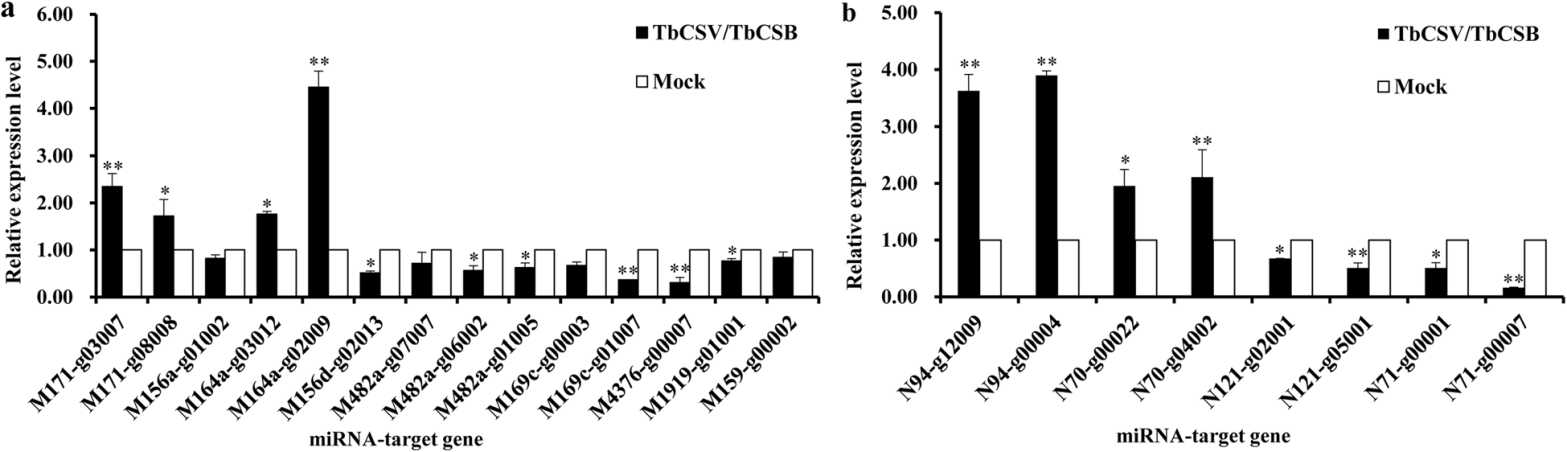
Relative expressions of miRNA-targeted genes. Relative expression of each gene was determined through qRT-PCR. **a** Relative expressions of the known miRNA-targeted genes. **b** Relative expressions of the novel miRNA-targeted genes. The x-axis shows the names of the miRNA-targeted genes analyzed in this study. The y-axis shows the Log_2_ratio between the expression values from a TbCSV/TbCSB-infected sample versus its Mock sample. Three biological replicates were analyzed for each miRNA-targeted gene through qRT-PCR. Expression level of *N. benthamiana UBC* gene was used as the reference gene during qRT-PCR assays. Asterisks indicate statistically significant differences compared with Mock, “*” indicate significant difference (0.01 ≤ *p* < 0.05), “**” indicate extremely significant difference (*P* < 0.01)

To validate the high-throughput sequencing results, nine known and four novel miRNAs that showed differential expression between the two libraries were selected and analyzed for expression by stem-loop qRT-PCR (Table 4 and Fig. 6). The PCR primers are listed in Table S1. The expressions of miR156d-5p, miR169c, miR4376, miR156a, miR1919c-5p, miR159, novel-121, novel-71, and miR482a in the infected library were all upregulated, whereas the expressions of miR171b, miR164a-5p, novel-94, and novel-70 were all downregulated. To examine if the expressions of TbCSV/TbCSB infection-regulated miRNAs could influence the expressions of their target genes, we analyzed the predicted target genes through qRT-PCR. The expressions of squamosa promoter-binding-like protein (targeted by miR156a), disease resistance protein (targeted by miR482a), nuclear transcription factor Y subunit (targeted by miR169c), calcium-transporting ATPase (targeted by miR4376), conserved hypothetical protein (targeted by miR4376), MYB-like transcription factor (targeted by miR159), heavy metal transport (targeted by novel-121), and N-acetyltransferase (targeted by novel-71) were all downregulated after TbCSV/TbCSB infection. By contrast, GRAS family transcription factor (targeted by miR171), NAC domain-containing protein (targeted by miR164a), transcription factor (targeted by novel-70), and 1-aminocyclopropane-1-carboxylate oxidase (targeted by novel-94) were all upregulated after TbCSV/TbCSB co-infection (Table 4 and Fig. 7).

**Table 4:** Expression of differentially expressed miRNAs and targeted mRNA genes analyzed

ID miRNA	log_2_(TbCSV/TbCSB / Mock)	qRT-PCR	ID target gene	qRT-PCR	Functional annotation
miR156a	1.27(up)	4.47(up)	Niben101Scf19266g01002	0.82(down)	Squamosa promoter-binding protein
miR156d-5p	1.05(up)	7.97(up)	Niben101Scf10743g02013	0.51(down)	Squamosa promoter-binding-like protein
miR164a-5p	−1.05(down)	−0.36(down)	Niben101Scf02318g03012	1.76(up)	NAC domain-containing protein
			Niben101Scf04745g02009	4.45(up)	NAC domain-containing protein
miR169c	1.23(up)	2.95(up)	Niben101Scf15723g00003	0.67(down)	YA2
			Niben101Scf10191g01007	0.36(down)	Nuclear transcription factor Y subunit A
miR171b	−1.15 (down)	−0.79(down)	Niben101Scf03072g03007	2.35(up)	GRAS family transcription factor
			Niben101Scf03693g08008	1.72(up)	GRAS family transcription factor
miR1919c-5p	1.10(up)	1.34(up)	Niben101Scf02655g01001	0.76(down)	conserved hypothetical protein
miR4376	1.39(up)	2.41(up)	Niben101Scf04808g00007	0.30(down)	calcium-transporting ATPase
miR482a	1.24 (up)	5.34(up)	Niben101Scf01683g07007	0.71(down)	Cyclin-dependent kinase
			Niben101Scf01052g06002	0.56(down)	Disease resistance protein (NB-ARC)
			Niben101Scf01941g01005	0.62(down)	Disease resistance protein (NB-ARC)
miR159	0.36(up)	2.42(up)	Niben101Scf11569g00002	0.84(down)	MYB-like transcription factor
novel 121	1.53(up)	2.55(up)	Niben101Scf00504g02001	0.68(down)	Heavy metal transport
			Niben101Scf02139g05001	0.51(down)	Heavy metal transport
novel 70	−1.91(down)	−0.12(down)	Niben101Scf02825g00022	1.96(up)	fiber protein Fb11
			Niben101Scf00578g04002	2.11(up)	Transcription factor
novel 71	1.61 (up)	6.50(up)	Niben101Scf12157g00001	0.51(down)	N-acetyltransferase 10 homolog
			Niben101Scf03820g00007	0.16(down)	N-acetyltransferase 10 homolog
novel 94	−1.93(down)	−0.25(down)	Niben101Scf19336g00004	3.90(up)	1-aminocyclopropane-1-carboxylate oxidase
			Niben101Scf04528g12009	3.62(up)	GRAS family transcription factor

## Discussion

High-throughput sequencing technology has been used extensively in small RNA research [45]. More and more studies have illustrated that various virus infections in plants often alter miRNA expressions [17–20, 46]. A large number of miRNAs have been identified in plants and the functions of many miRNAs have also been investigated [47, 48]. To better understand the roles of the *N. benthamiana* miRNAs in host resistance to TbCSV/TbCSB co-infection, in this study, two libraries were constructed, using total RNA from *N. benthamiana* plants either infected with TbCSV/TbCSB or not.

The most abundant small RNA reads in the two libraries were those with 24 nt. In addition, the 24 nt small RNA reads were the predominant unique small RNA reads. This finding agrees with earlier reports which have shown that 24 nt small RNAs are more abundant in several other diseased plants, such as tomato plants infected with *Phytophthora infestans*, tomato infected with cucumber mosaic virus (CMV) and wheat plants infected with powdery mildew pathogen [13, 49, 50]. The length distribution of small RNA reads may reflect their compositions [51]. We found more 24 nt small RNAs in the non-infected library than in the infected library. By contrast, 21 and 22 nt miRNAs were more abundant in the infected library. Our results also indicate that the expression profiles of miRNAs were significantly altered after TbCSV/TbCSB co-infection in *N. benthamiana*, and the differentially regulated expressions of miRNAs suggested that miRNAs play important roles during TbCSV/TbCSB co-infection.

In all, 13 known and 42 potentially novel miRNAs were differentially regulated by TbCSV/TbCSB co-infection. To better understand the relative abundances of miRNAs in the two libraries, we analyzed sequence frequencies and used them as indexes. When the two libraries were compared, the normalized reads varied from about 2 (novel-96) to 63,693 (miR482a) in the infected library and from 0 (novel-131) to 28,999 (miR482a) in the non-infected control library, indicating significant variation in the relative abundances of different miRNA sequences. This finding was later confirmed through qRT-PCR analyses using several selected differentially expressed miRNAs.

miRNAs may regulate host defenses against pathogens, including viruses, by suppressing pathogen multiplication at the post-transcriptional level [13, 52]. Several stress-responsive miRNAs (e.g., miR168, miR169, and miR482) have been reported to target transcription factors controlling host resistance to virus infection [53–55]. In *N. benthamiana*, virus infection may regulate the expression of miR168 to alleviate the anti-viral function of AGO1 protein [53]. In our study, *N. benthamiana* miR168 was found to be responsive to TbCSV/TbCSB co-infection. In addition, the expression of miR169 was upregulated after co-infection. In a previous study, rice miR169 was found to negatively regulate rice immunity against *Magnaporthe oryzae* infection by differentially repressing its target genes [54]. Studies also showed that rice miR164 plays an important role in rice resistance to southern rice black-streaked dwarf virus (SRBSDV) infection as well as rice resistance to drought stresses by differentially regulating its target genes [56, 57]. In addition, miR482 can regulate the expression of *NBS-LRR* defense genes during fungal pathogen infection in cotton [55]. In our study, when the expressions of miR164a and miR482 in the two libraries were compared, the normalized miRNA164a and miR482 reads were 3624 and 28,999 in the non-infected control library, and 1633 and 63,693 in the infected library, respectively, suggesting that these two miRNAs may play roles in *N. benthamiana* resistance to TbCSV/TbCSB co-infection. The further studies will be continued to unravel the functions of these miRNAs.

## Conclusion

In this study, miRNAs regulated by TbCSV and TbCSB co-infection in *N. benthamiana* were characterized using high-throughput sequencing technology, and some miRNAs involved in plant defense system were found to be significantly regulated after TbCSV and TbCSB infection. The molecular functions of these miRNAs in *N. benthamiana* resistance to TbCSV/TbCSB co-infection may require further investigation. Nonetheless, our results improve knowledge of the infection of TbCSV/TbCSB in host plants while also providing additional information for the development of management strategies for TbCSV/TbCSB infection in the future.

## Supplementary information

**Additional file 1: Table S1.** Primers used for qRT-PCR.

**Additional file 2: Figure S1.** The precursors of 42 novel microRNAs and their hairpin structures in *N. benthamiana.*

**Additional file 3: Figure S2.** First nucleotide bias and nucleotide bias analysis.

**Additional file 4: Table S2.** 42differentially-expressed novel miRNAs and 25 equally-expressed novel miRNAs with miRNAs in *N. benthamiana.*

## Data Availability

All data and materials described in the manuscript are available in the Additional files 2,3 and 4.

## Abbreviations

miRNA: MicroRNA
nt: Nucleotide
qRT-PCR: Quantitative real time polymerase chain reaction
TbCSB: Tobacco curly shoot betasatellite
TbCSV: Tobacco curly shoot virus
TPM: Transcripts per million reads
