# Supplementary material for: Identification of microRNAs regulated by tobacco curly shoot virus co-infection with its betasatellite in Nicotiana benthamiana
Source: Virol J. 2019 Nov 7;16:130. doi: 10.1186/s12985-019-1234-5 (PMC6836351; doi:10.1186/s12985-019-1234-5)
Supplement: Supplementary file 1 — Additional file 1: Table S1. Primers used for qRT-PCR. [file 12985_2019_1234_MOESM1_ESM.docx]

**Table S1** RT-qPCR primers for miRNAs and mRNAs.

**RT-qPCR primers for miRNAs**

| **miRNA primers** | **Primer sequence (5’-3’)** |  |
| --- | --- | --- |
| miR156a-RT | GTTGGCTCTGGTGCAGGGTCCGAGGTATTCGCACCAGAGCCAACGTGCTCT |  |
| miR156a-F | CGCGCGGTTGACAGAAGATAG |  |
| miR156d-5p-RT | GTTGGCTCTGGTGCAGGGTCCGAGGTATTCGCACCAGAGCCAACGTGCTCA |  |
| miR156d-5p-F | CGCGCGGTGACAGAAGAGAG |  |
| miR164a-5p-RT | GTTGGCTCTGGTGCAGGGTCCGAGGTATTCGCACCAGAGCCAACTGCACGT |  |
| miR164a-5p-F | CGATCAGTGGAGAAGCAGGGC |  |
| miR169c-RT | GTTGGCTCTGGTGCAGGGTCCGAGGTATTCGCACCAGAGCCAACTCGGCAA |  |
| miR169c-F | CGCACGTCAGCCAAGGATGAC |  |
| miR171b-RT | GTTGGCTCTGGTGCAGGGTCCGAGGTATTCGCACCAGAGCCAACCGTGATA |  |
| miR171b-F | CGAGCAGTTGAGCCGTGCCAA |  |
| miR1919c-5p-RT | GTTGGCTCTGGTGCAGGGTCCGAGGTATTCGCACCAGAGCCAACGGGCGAA |  |
| miR1919c-5p-F | CGCGCAGTGTCGCAGATGACT |  |
| miR4376-RT | GTTGGCTCTGGTGCAGGGTCCGAGGTATTCGCACCAGAGCCAACTCCAGCA |  |
| miR4376-F | CGCTCGACGCAGGAGAGATGA |  |
| miR482a-RT | GTTGGCTCTGGTGCAGGGTCCGAGGTATTCGCACCAGAGCCAACTAGGAAT |  |
| miR482a-F | CGCGCGTTTCCAATTCCACCC |  |
| Universal-R2 | GGTGCAGGGTCCGAGGTAT |  |
| Nb-UBC--F | TTTCGGTCCTGATGATACTCCC |  |
| Nb-UBC--R | CACAGAGCAAAGACTGGATTGA |  |

**RT-qPCR primers for targets (mRNAs)**

| **mRNA primers** | **Primer sequence (5’-3’)** |
| --- | --- |
| M171-g03007-F | TCATTCGTTTCGCTGAGTGC |
| M171-g03007-R | CGTTGAAGAGGTTTTCCGGC |
| M171-g08008-F | TAACGCCCTTGATCTCTCCG |
| M171-g08008-R | AGCGAGTTGGGCTTGTAGTG |
| M156a-g01002-F | GACTGATGCACAGCGAATCC |
| M156a-g01002-R | TACTGGCACAGACAATTCAGGG |
| M156d-g02013-F | GCAGGAGGAGATTAGCAGGAC |
| M156d-g02013-R | TGGGAGATCGCCCTTTGAAAT |
| M482a-g07007-F | ATTAGGGAGCAAGAAGGGAATGG |
| M482a-g07007-R | GCCATCCATCCACCAATTCATC |
| M482a-g06002-F | ATCCAGAAAATGTCGTCGTC |
| M482a-g06002-R | ACATGGATGTCAAAGCGATGC |
| M482a-g01005-F | AGCAGGGGAGTTCTTACTTGG |
| M482a-g01005-R | CCAAGCACGGATGTCAAAATG |
| M159-g00002-F | TCCTCGGGTGTAATTCCTGGC |
| M159-g00002-R | AAGATAAGCCCCAACTTGCC |
| M169c-g00003-F | GGCAGTGCCAACATAGACTG |
| M169c-g00003-R | ATGACGTGCAGAACCAACCT |
| M169c-g01007-F | TTATCCATGATGTCGGTGCC |
| M169c-g01007-R | ATGACGTGCAGAACCAACC |
| M4376-g00007-F | AGAAGAAACAAGGGAGACGAGG |
| M4376-g00007-R | TCTTCGCCACTCATCTTCTG |
| M1919-g01001-F | AGAAATACGAGTGTGGGTTCC |
| M1919-g01001-R | AGGCTTTAGACTTTACTCCTTTCC |
| M164a-g03012-F | CTTCCACCGCTCCTTGATTCT |
| M164a-g03012-R | CCAAAGACAGAACTTGGGTCG |
| M164a-g02009-F | TTCCACCGCTCCTTGATTCC |
| M164a-g02009-R | GCATGCAGGCTGTTCGATG |
| N121-g02001-F | TCATACGTGCCACCATCACC |
| N121-g02001-R | TCTCATTCGACCGGTGAAACT |
| N121-g05001-F | GTCACCACCACCAACGTACT |
| N121-g05001-R | AGTTACCCAGACGTTCTTTGAG |
| N71-g00001-F | AGATAGAATTGAGCGAATCC |
| N71-g00001-R | AACATAGAGGGCCATTCGCT |
| N71-g00007-F | AATGCACTTTCAGGTCGGCT |
| N71-g00007-R | AAGAGAAAAGTGTGTCCCGG |
| N94-g00004-F | ATGAAGGCCATGGAATCTGCT |
| N94-g00004-R | AAGATGCAGAGACAACCCCTT |
| N94-g12009-F | CCGGCTGGAAATGATTTGCTC |
| N94-g12009-R | GCTTCTGCTATGGCACCATTT |
| N70-g00022-F | ACTCTGCAAATACCATCCGTCG |
| N70-g00022-R | AGTGGACGTCGCATAACTTTC |
| N70-g04002-F | ATTGAGGGGTGAAAAGGCTGA |
| N70-g04002-R | GAGCTGCTGATTCTTGGCTTA |
