# Supplementary material for: Identification of microRNAs regulated by tobacco curly shoot virus co-infection with its betasatellite in Nicotiana benthamiana
Source: Virol J. 2019 Nov 7;16:130. doi: 10.1186/s12985-019-1234-5 (PMC6836351; doi:10.1186/s12985-019-1234-5)
Supplement: Supplementary file 2 — Additional file 2: Figure S1. The precursors of 42 novel microRNAs and their hairpin structures in N. benthamiana. [file 12985_2019_1234_MOESM2_ESM.docx]

**Figure S1** The precursors of 42 novel microRNAs and their hairpin structures in *N. benthamiana.*


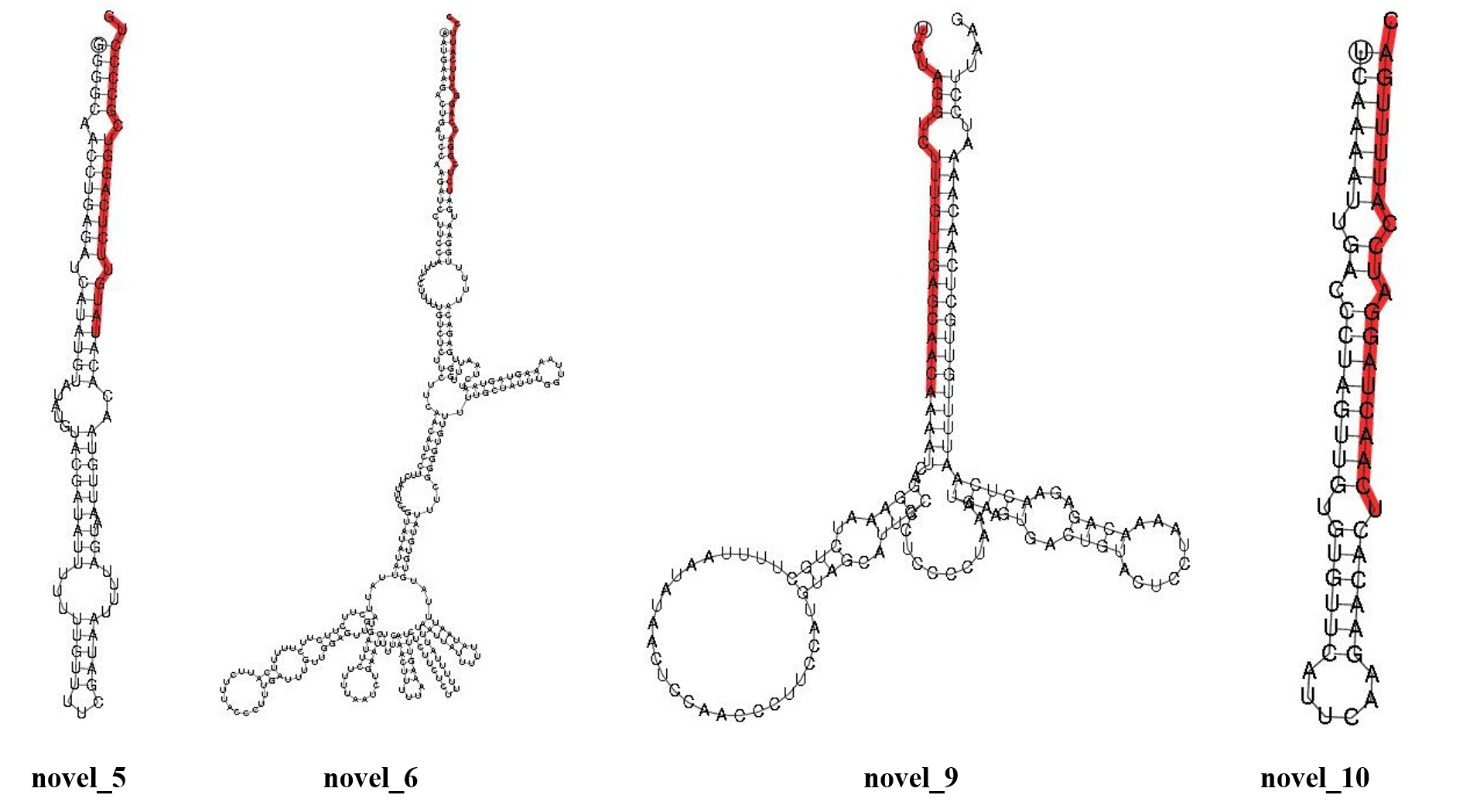

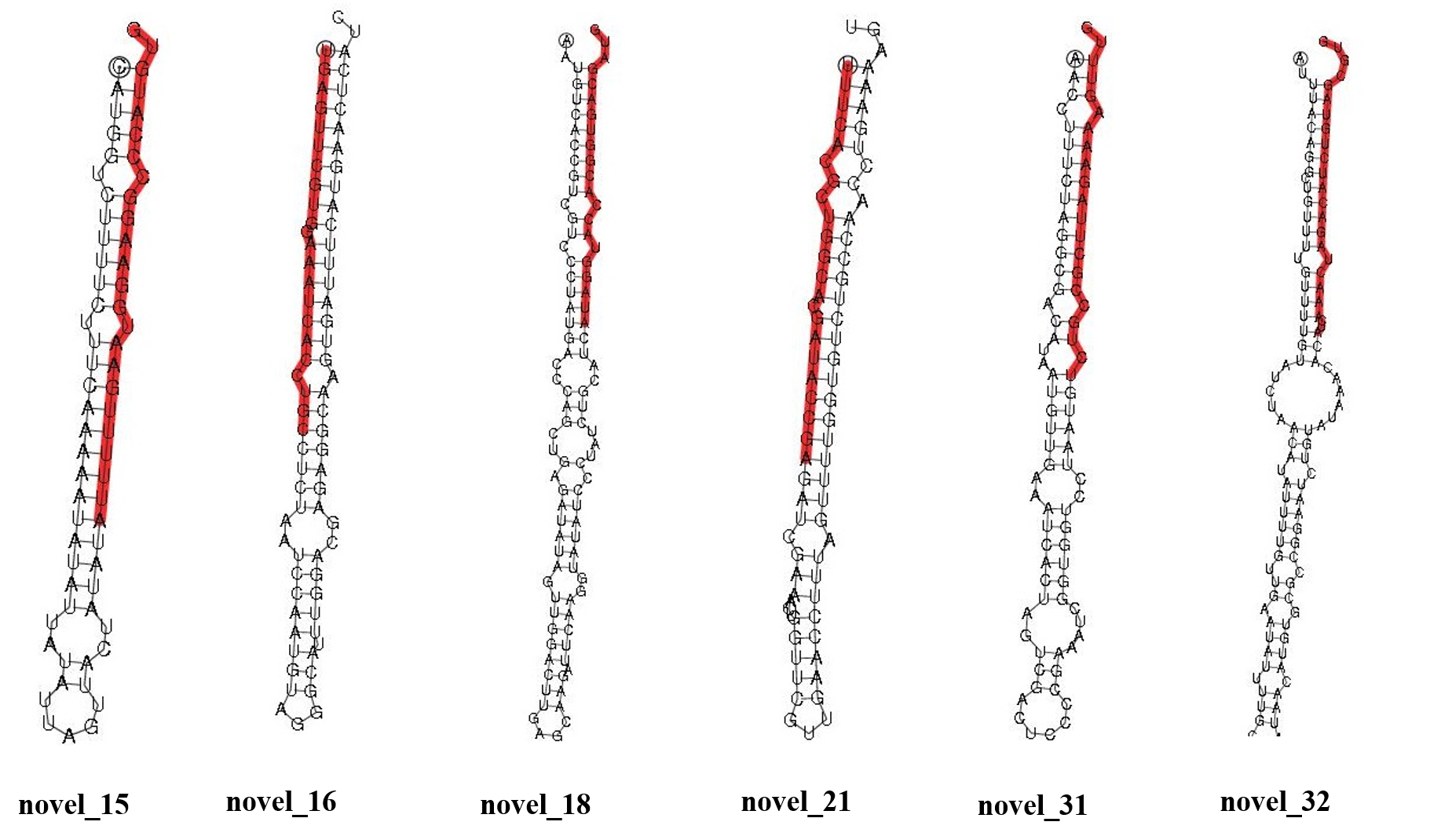

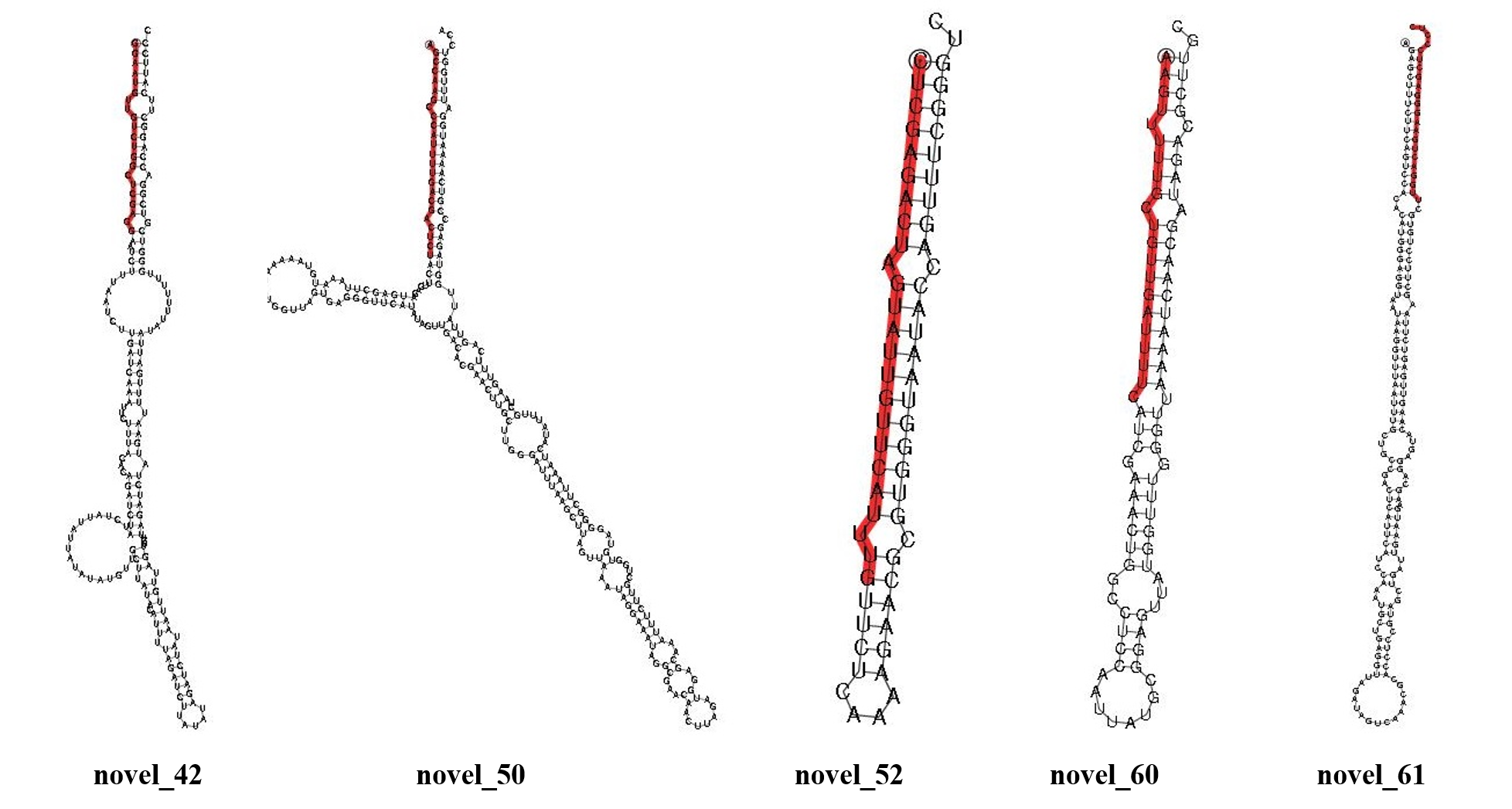

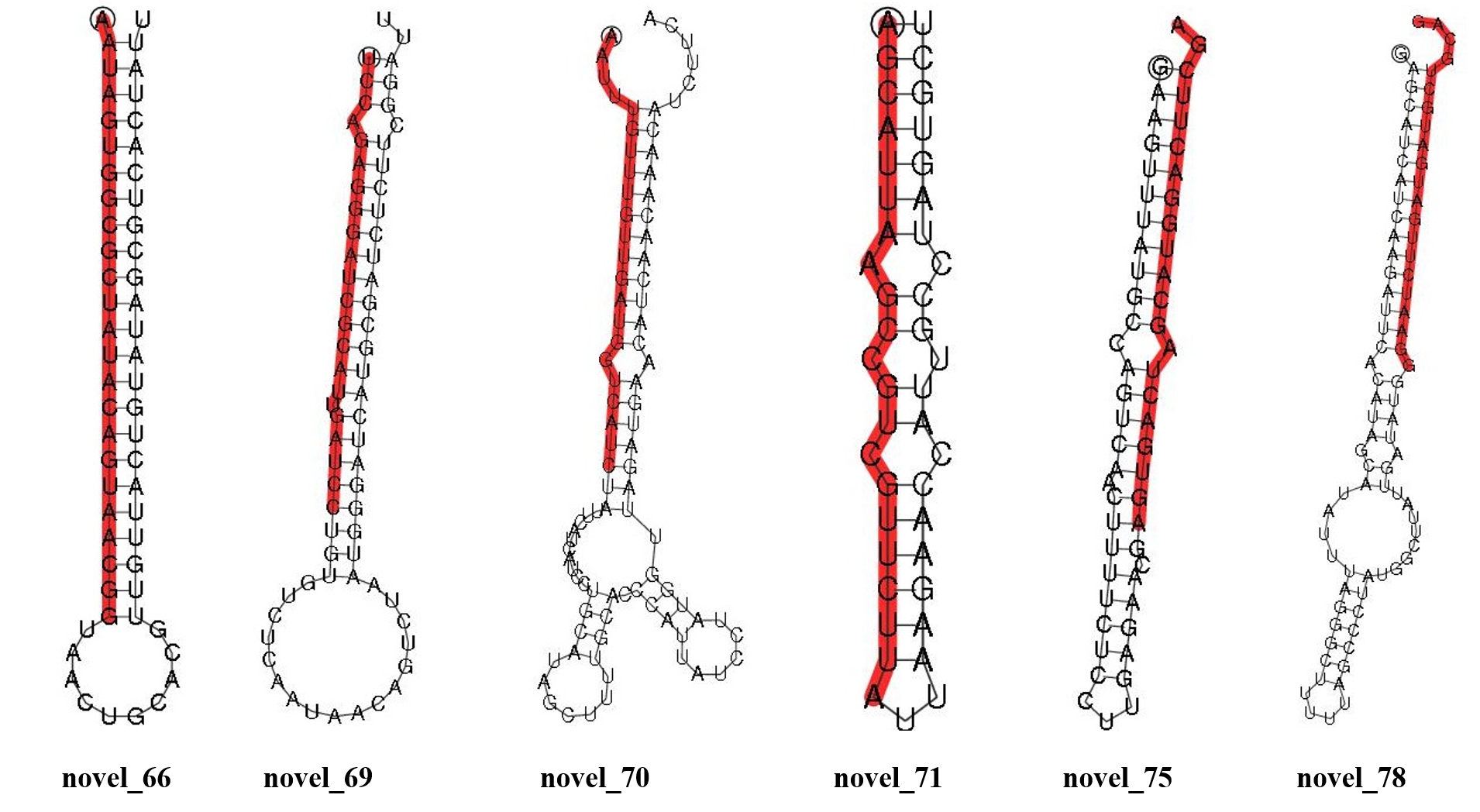

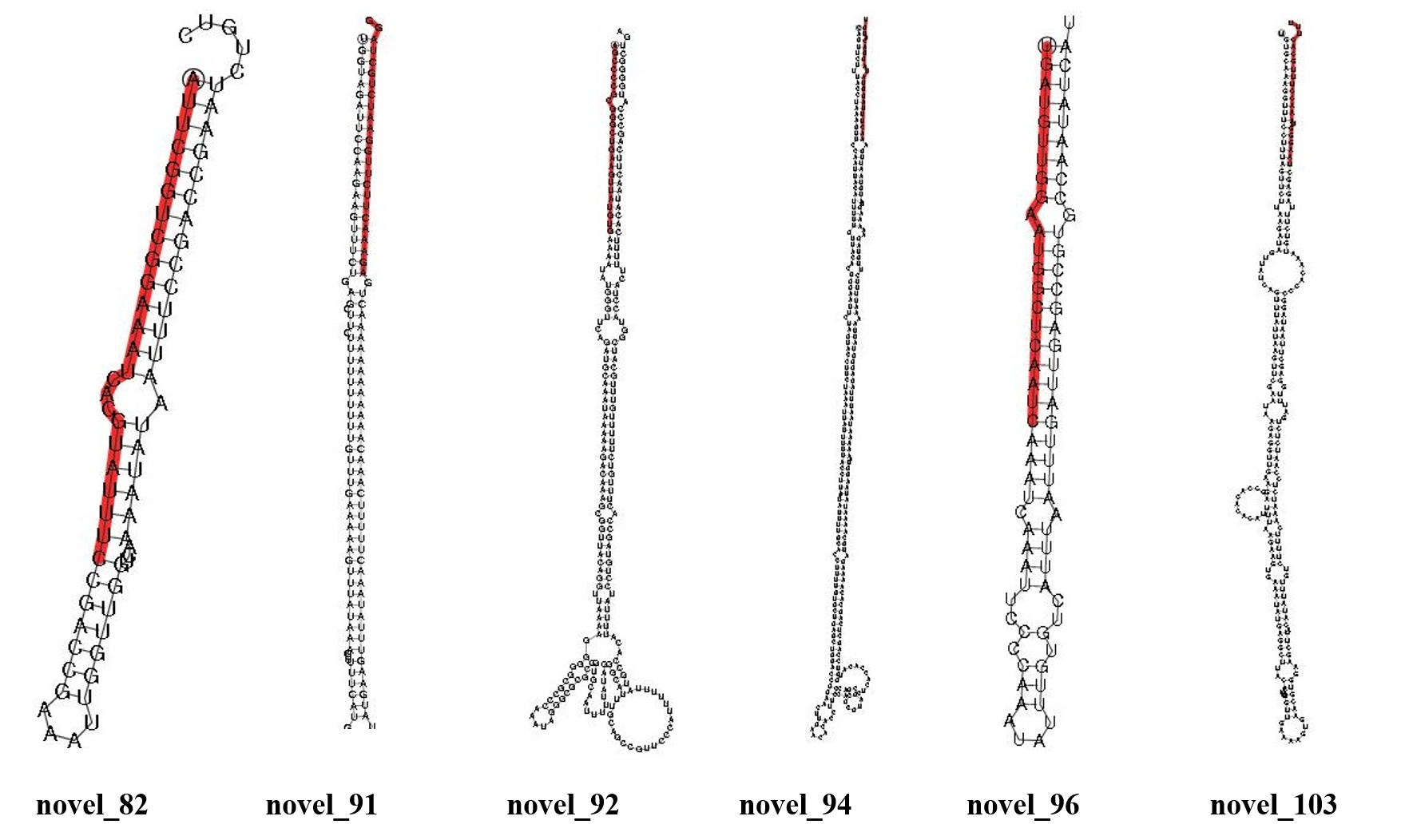

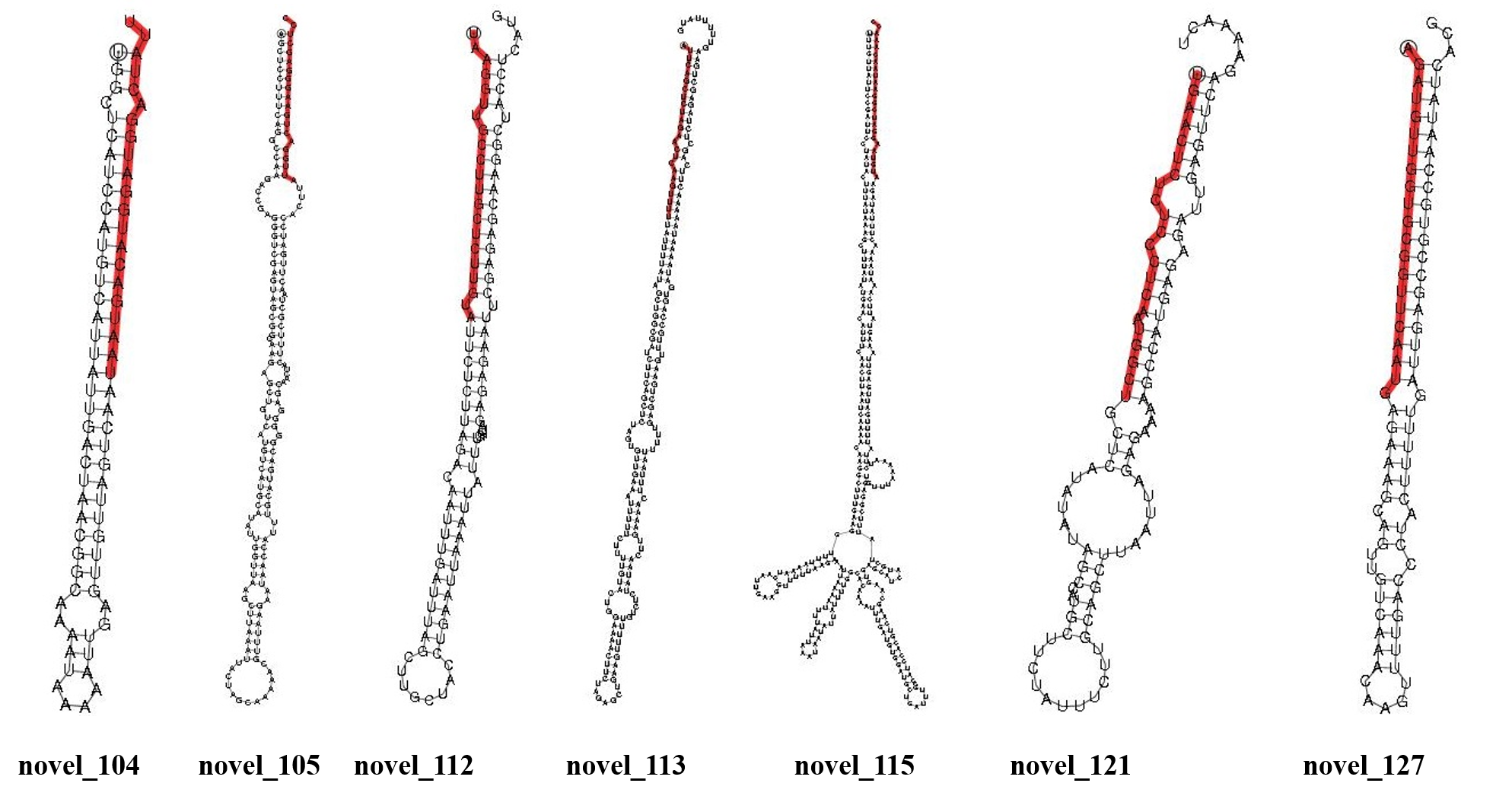

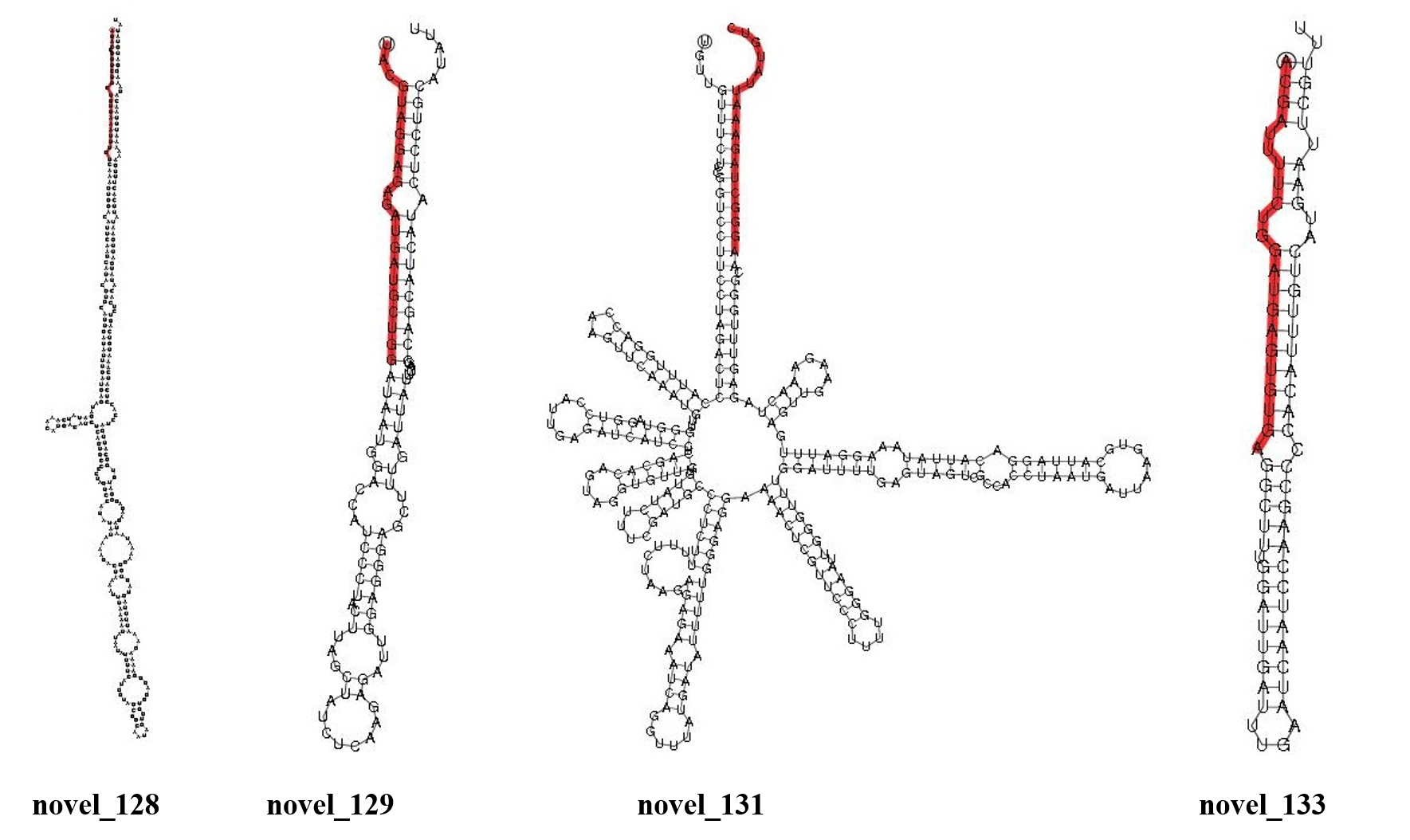

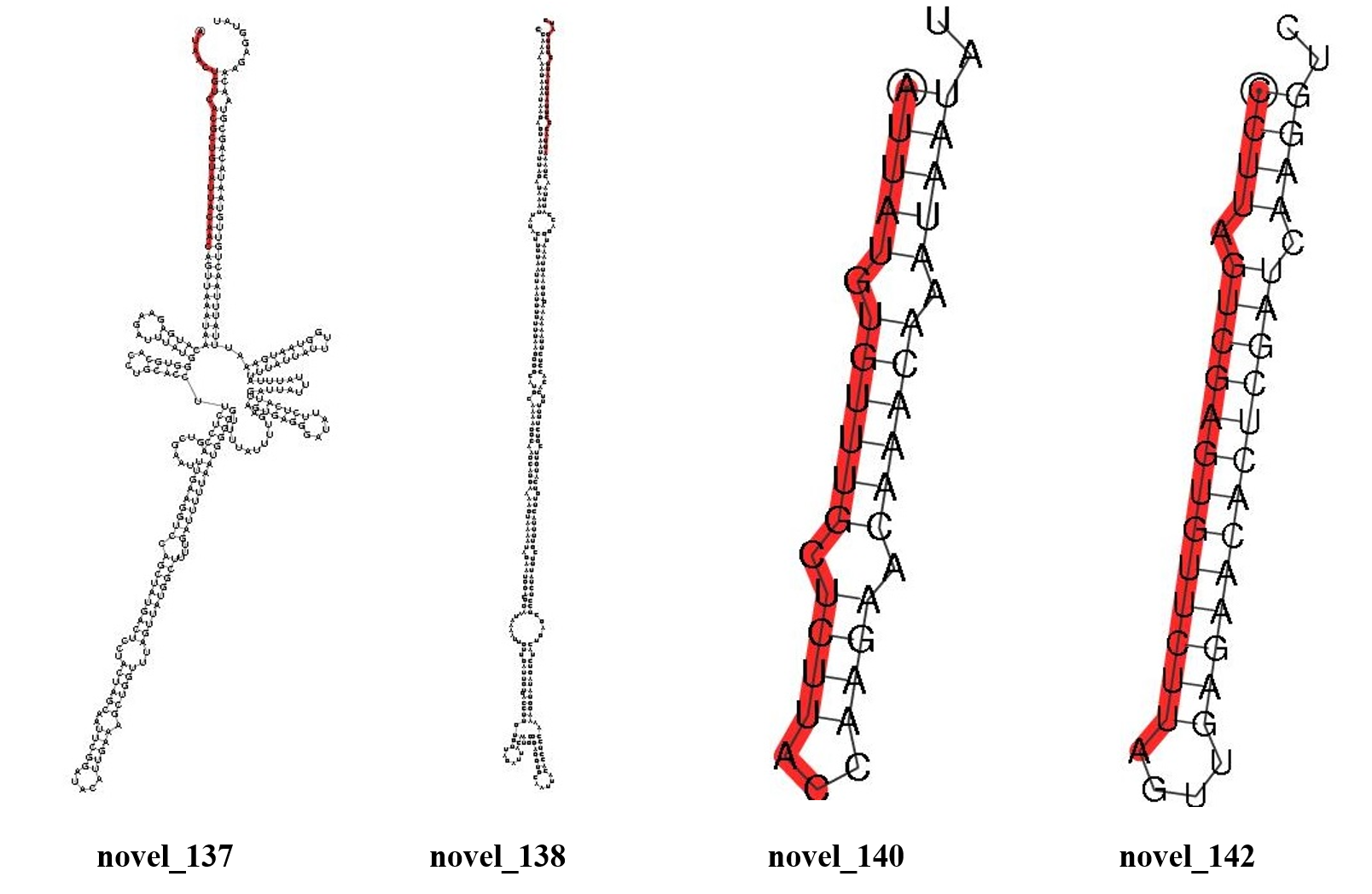


**Novel-10**

UCAAAAUUGACCCUAGUUGUGUGUUCAUUCAAGAACACUCAACUAGGAUCCAUUUUGAC

**Novel-103**

UGUGCAAAGGUUUCCUUUAGUUCUUAAGAUAGUUAUCAGUUUAUUAAGUUCGAAUAAGAGGUUGAAGAGCCACACACAUUUUAAGAAGUGAAAUAUGAGGCUUACAAUUUGUUGAAAAGUGAACGUGGAAGCUUACAUAUUUGUCUUUUCAAAUCUCCAAUCUCUGAUUUGGAGCUUAAUAGGCCCACAAAUGUCUUUAGAGCUAAGGGUAAACCUUUGCACUUU

**Novel-104**

UGGCUCAUCCAUGUCAUUAUUGACUAACGGCAAAAUAAAAAUUGAGUUGUUAGUCAAUAAUGACAUGGAUGGACUAUU

**Novel-105**

AGCUCCUUUCAGGCCAAGACCGAGGGUCGAGUAGCGGCAAGAGCUGUCAUGUCAUGCAUAUUGGUUAAGCUUAAAUUACUAGCAAAAACGUUUAAGAAUAACCAUUUGCAUGACGGGGGAGCAACUACUUUCGCUAACUUGAUCCACUUAUUGGACUGAAGGGAGCUCC

**Novel-112**

UAAGGUUGCCUUGCUCUUGUAUUCUCUUAGACAAUUUGAUUUAGCUUGCUACCUGAAUUAAAUUAUUUGGUAGAGAGAAUUCGAGAGCAAGGCUACCUCAUG

**Novel-113**

AUUCAGCUCUAGAACUCAAGUUUUUAUUUUAUAGCUGGCGAUCUUCAGCUCUAGUGUUGAAAUUUUUCUUUUGUACUGGAAAACUUCUAGAGCUGAAGUUUUUGUCUCUAUAACUUGAAAACUUUAAUUUUUGAGCUGAAGUUUGCCAGUGAUAAAAUAAAAACUUCAGCUCUAGAGCUGAAGUUUUUAUG

**Novel-115**

UUUGUUAUUCCGAUUCCUAUACUUUAUAAAGCUUUAUAUGAACAUUUCAACUUAAUCAAAACAAGGCUUUGAAGGUUUUAAAAUCAAUUGAAGGUUUUUAAGAAAUUAAAAUUUUAUUAAAUAAUAUUAUUUUGGCACAAAAUUUGAUGUGGAUGCUGAUUUGCCAUCCACGUCAAGCAAGUGAGCAUCAUGCUAUUUCGGAGUGUUUAAAAAUCUUAUUUUGAUUGAGUUAAAGUAUUCAAAUAAAACUUUAUAGAAUGUAAGGAUCGGAAUAACAAACC

**Novel-121**

UGAACUCUCUCCCUCAAUGGCUGCUCAUAUAUAGCCAUGCUUCUAUUUCUUGCAGCUUAAUUAGAGAAAAGCCAUGAGAGAUUGAGUUCAGAAAACU

**Novel-127**

AGAUGUUGGUGCGGUUCAAUGAGAAAGCAGUUGUCAAACAAGUUUUGACCCUACUUUUUGAUUGAGCCGUGCCAAUAUCACG

**Novel-128**

AUACCGUUCUUCUGUUAAAUUUGUCAAAGUGGACAUUCAAUCAUACGUGCAUUGAUAUUUGAUGAGAUAGAUAUCAAACAUGACAUCUCAGUGCCCCUAGUCCAUAUAUAAAAGACUAAAAUUUAAAAUAUAAUAUUUUCAUGGUAGCGGCAAUAGUGGUGGAGGGAAAAGAAAUUUUAGUAGUGGGAAAAUAAUAGAAGAGGGAUGUGGCAUUGAUCCACCUCAUCAAAUGUCAGUCUCACAUAUGAUUGAAUAUUCACUUUGAAAAAAUUUAACAUAAAGGAUGUAUAU

**Novel-129**

UACGUAGGAGAGAUGAUGCUGGAUAAUGGACCAUCCCUACUUAGCUAUCUCAAGAGAUUGGAGGGAGCUUUGAUUAUUUAGCCAGCAUCAUACUCCUGCAUAUU

**Novel-131**

UGUUGUUUCUCCGGUCCUUCCUAGACUCCAUUUGGACCAAGUUCAAAUGUUGGGUAGGUCCAUUGAGAUCAUCCUCAGCACAGUAGGUGUUGUGUAUCUUUCGAUGCCCUCUUUUUCUAAGAGAGAAAUCAGGUUUAUGAUAUUUUUGGGAGGAAAACUCGUUCCCUUUUGGGAAUUGGGUUUGGAUUUUGAGUAGUCGCCACCUAAUGAUUAAGUGCAUUAGGACAUUAUAAAGGAUUUGAGUUGAAGAAACUAGAGUUUGGGCAAGGGCUAGAAAUUAUGUC

**Novel-133**

ACGAUUUUCUGGAUGAGUGUGAGGCUUUGGAUUGAUUUGAAUCAAUCCAAGCCCCACAUUUGUCAUGAAUUCGUUU

**Novel-137**

AUAACUGUCACGCUGUAUUACAACAGUUAAAUAACAUGAGAAGAUUUAUGGGUGCACCUGCACCUUCUCCAGUCGAAUUUGAAGGUCCAGCUAUGACUCCUACUAGCAAUUCGGAUACAUUGAAAGCUGGUUUAGUUAUGGCUUUUGAUUUUUAAUGGGGGUUUUAUUUUGAAGUGAGGGAUAUUCUCAUUGAUUAUUUAUUUAUUUAUUAUUUGGUAAUGAAAUUUAUUUAACUGUUGUAAUACAGCGUAACAAGAGGUAU

**Novel-138**

CCAAAAAAUAAAUAAGAGUAAUUUAGAUAAAUUAUACUUUUAAUUAAUUUUUUUAAGGGGCAUGCAAAAGGCAAGCAUGAAAAGUAAAAUAGAAUGGACGGAAUAAAAUUGUUGAUUGUUACCUUGUGUUAGAUUACAAUGCGGAGGUGCAAUUACACCUCCAAAAGGUAUAGUCUACUGAGCUCCUCUAUUUCGUUUUACUUGUCAUGUUCGUCUUUUUUCACACCUCUUAAAAAAUAUUAAUUAAAUGGACCAUUUAACUAAAUUACCCUUAUUUAUUCUUUGAUC

**Novel-140**

AUUAUGUGUUUGCUCUUACCAAGAACAAACAAAUAAUAU

**Novel-142**

CCUUAGUCGAGUGUUCUUAGUUGAGAACACUCGAUCAAGGUC

**Novel-15**

UGAGUUCGUGGAAAUCACCUGCCUCUAAUCCAAUGUAGGGCAUUUGGACGAGAGGCAAGUGAUUUCAUGAACUCAUC

**Novel-16**

AAUGUCACCGUCGUCCCUAUGACCCAGCUGAGAUAUAGUUGGACUUGAGCAAGAUUCAAGGUAUAUCCCUAUCUGCAUCAUAGGUACCACGGUGACGAUG

**Novel-18**

UUUCACGCUGGCACGAUACCGAGAUCGAAACUGGUUCGUUGAACCUUUAGUUUUGGUGUCUGCCAACCUGAAAAGU

**Novel-21**

AACCUUUCUAGGCGACAUAAUGUUGAAAUCACUAGUCGACUCCCCGAAAUCGGUGGUCCUAAUGUCUGCCGCUUAGAAAAGUUUG

**Novel-31**

CAUGGUCUUUUCUUUCAAAAAUAUAUUAUAUUAGUUACUAUAUAUUUUUGAAUGGAAGGCCCAUGUG

**Novel-32**

AUUUACAGGCUGUUUUGUUUUGUAUCUAACAUAUUUUGUUGAAUAUUUUGCUAUAACAUGUGCGCCGGAAUCUGUAUAAACACAUCAAACUAGACAUCUGUAGCGUG

**Novel-42**

GGAAUGUUGUCUGGCUCGAGGAAUCUUUAAUCUUGAUCAAAUUCUUUACACAGAUCUAUCUAUUAUUAUAUAUGUUGCUUAUACAUUUUAGAUCUUAUAUAGAUCUAUAAUUGUUAGUUGUUAGAUCUAUGAAUUUUGAUUAUAUUUUUUUGGGUCGUCGGACCAGGCUUCAUUCCC

**Novel-5**

GGGGCAACCUGAGAUCAUAUGUAUAUGUACGAUAUUUUUUUGUUUUCGAUAAUUUUAGUAAUUGUAACACAUAUGUUCUCAGGUCGCCCCUG

**Novel-50**

AGCCAAGCCCAUUUUGACGACUCUACUUGAGAUGAGCUUAAAUGUAAAAAUAUGGUUAGUGAGGGUUCAUAUAGUUGACACGAACUUGCUUGGGAUUUAAGCUUAGUUAAAUAGGAAAUAGGCGAACAACUUAGAUGGAGCAAAUUUCUUGCUGGUGUAGGGGCUUAAAUCAUAUUUGCUAAGUUUCAGUUAUUGGUAGAGCCGUCAAAAUGGAUUUGGUCCA

**Novel-52**

CUCGAGACUAGUAUUGUUCAUUUGUUCUCAAAAGAACGCGUGGGUAAUACCAGUUUCGGGUC

**Novel-6**

AAUGAAGACUGAUCCAAGAUCCUUCCAUUUCCUUUUUGUCUCUUCUUCAACAUCCUUCUAUUUCCGUAUAUAUUAUUCUUCUUCUUCUUUUUCAUUCUUUACCCUUUGAUUUGUUGGAGUUGAUGAUUCUUUUAAUCUGAAUCUUUUCAAACUUUUUAAAGUUUCUCUUCUCUUUUUUAUUUAAUUAUUUUUAUAAUUUAUGUGUGUAUUUCGGGGUGUUUUUGCUAUUUGGUUAAAAGUAGUAAUCUAAUUUGGUGAGACAUUUUUGGAAUGAUCUCGGACCAGGCUUCAUUCC

**Novel-60**

AAGUUUUUGCUGUUGAUUUUCAUCGAAACUGGCCUCCAAUUAUGCGGAGUUAUGGUUUGGGUUAAAAUCAACGAUAGACGCUUGC

**Novel-61**

AGAGCUUUCUUCAGUCCACACAUGGGAGGUAAUAAGGUUUAAUUUGCUGCCGACUCAUUCAUCCAAAUGCUGAGGUUAGAUAGUCAAACGCACCUCCGUAGCUGAUUGAAUGAAGCAGGAGUACAAGUUGAGUCUUAAGCUUCCUGUGCUUGGACUGAAGGGAGCUCCCUC

**Novel-66**

AAUAGUGGCGCUAUACAGUAACGGUAACUGCACGUUGUUACUGUAUAGCGUCACUAUU

**Novel-69**

UCCAGAGGGAUCGCAUUGAUCCUGUGUCUCAAUAACAGUCUAAUGGGAUCAUGCGAUCUCUUCGGAUU

**Novel-70**

AAUUUGUUUGUUGAUGGUCAUCUAUUCAUCAUCCUGCAUAGCUUUUGCACCCAUUAUCCUAUGGUUAGAUGAACAUCAACAAACAUCUUCA

**Novel-71**

AGCAUUAAGCCGUCGUUCUUAUUAAGAACCAUUGCCUAGUGCU

**Novel-75**

GAAGUUUAUGCCAGUCAACUUUUCUCCUUGAGAACGAGUGACUAGCAUGGACUUCGA

**Novel-78**

GAGCAUCAUCAAGAUUCACAUAGCAUAUUUAGGGCUUUUUAGCCCUAUGGCUUAUUGAUAUGGGAAUCUUGAUGAUGCUGCAG

**Novel-82**

AUUCGGUCGGAAAUCACGUAUUUCCGACCGAAAUUGGUUGGUCAAAAUAUAAUUUCCGACCGAAUCUGUC

**Novel-9**

UCUAGGUCUUUGUUGAGCAACAAAAUCAGGAAAUCUGCUUUUAAUAUAACUCCAACCCUUCCAUGUAGCAUUCCCCUCCCCUAAAUGAAGUGACUGUACUCCUAAAACAGAGAACUCAAUUUUGUUGCUCAACAAAAUCCUUAAG

**Novel-91**

UGGUAGAUUCCAAGAAGUUUCUGAGCUUCUUUUUUUUUUGUUUGAAAAAGUUUAUAAAGUUUUUCAUGUUUAUGAAGUUUAUAAACUUUUUCAAACAAAAAAAAAAAACUGAGAAACUUCUUGGAAUCUGCUAGG

**Novel-92**

AGCCCCGCGGGCUGAAGUUAUGUGAAAAUAUGGGUUCAGAUGCAAAUAAAAAGACAAAGCGGUUACAGGUUAAAAGGGGGCGCCCAAAUAGGGCGCGCCGUGCAAUUUUUAUAGGGCAUUUGCAGCCGUUCCCAUUUUUUAUGCCACAUUUUAUCCUGUAGCCACUUUGUCUUUUUGUUUGCAUCGGUACCUACUUUUUCACAUAACUUCAGCCCAUGGGGCUGA

**Novel-94**

CAGUUCUUUACCUAAAGUUCCAAUUACAUUUUUGUUACACGGGAAUUCUAGUACCUUCUAAAUUAUUUUUACCUUAAUAUUUUGCACCUUUUGUGCUGAGCUGGACCGGAACUUGAACACACCUUCCCCAAGCGCGUAGCCUCACCACAUGGUCCAGCUCAGCACAAAAGAUGCAAAAUAUAAGGUAAAAAAUAAUUUAGAGGGUAUUAAAAUUUUCUUGUAGAAAAAGUAUGUAAUUGAAACUUUAGGUAUAGGACAUGU

**Novel-96**

UGAUGUUGGAAUGGCUCAAUCAAAUCAAAUUCCCCAAAUAUUUGUGUCAUUUAAUUUGAUUGAGCCGUGCCAAUAUCAU
