# Supplementary material for: Identification of microRNAs regulated by tobacco curly shoot virus co-infection with its betasatellite in Nicotiana benthamiana
Source: Virol J. 2019 Nov 7;16:130. doi: 10.1186/s12985-019-1234-5 (PMC6836351; doi:10.1186/s12985-019-1234-5)
Supplement: Supplementary file 3 — Additional file 3: Figure S2. First nucleotide bias and nucleotide bias analysis. [file 12985_2019_1234_MOESM3_ESM.docx]

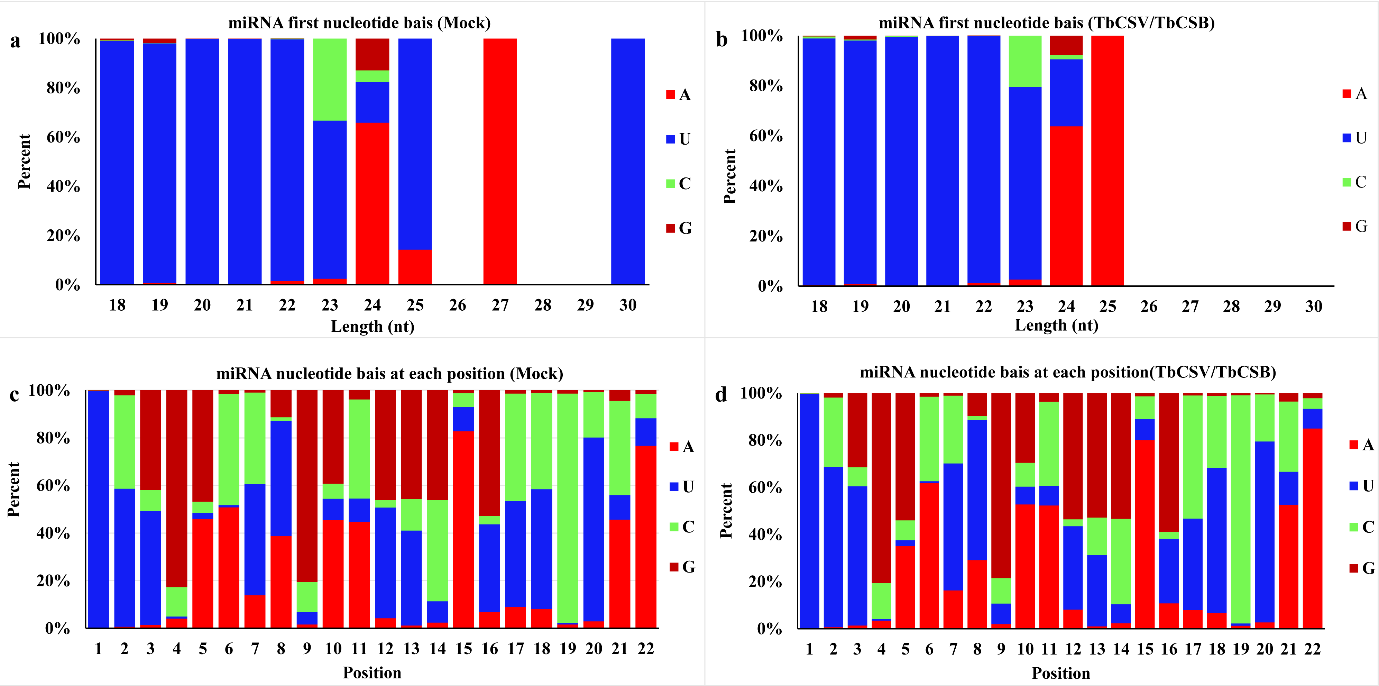


**Figure S2** First nucleotide bias and nucleotide bias at each position in novel miRNA candidates from the non-infected (Mock) (a, c) and the TbCSV/TbCSB-infected (b, d) *N. benthamiana* libraries, respectively.
